# Molecular Mechanism by Which the GATA Transcription Factor CcNsdD2 Regulates the Developmental Fate of *Coprinopsis cinerea* under Dark or Light Conditions

**DOI:** 10.1128/mbio.03626-21

**Published:** 2022-02-01

**Authors:** Cuicui Liu, Liqin Kang, Miao Lin, Jingjing Bi, Zhonghua Liu, Sheng Yuan

**Affiliations:** a Jiangsu Key Laboratory for Microbes and Microbial Functional Genomics, Jiangsu Engineering and Technology Research Center for Industrialization of Microbial Resources, College of Life Science, Nanjing Normal University, Nanjing, People’s Republic of China; b College of Life Science and Chemistry, Jiangsu Second Normal University, Nanjing, People’s Republic of China; University of British Columbia

**Keywords:** transcription factor, fruiting body, primary hyphal knot, secondary hyphal knot, primordium, sclerotium, light stimulation, *Coprinopsis cinerea*

## Abstract

Coprinopsis cinerea has seven homologs of the Aspergillus nidulans transcription factor NsdD. Of these, CcNsdD1 and CcNsdD2 from *C. cinerea* show the best identities of 62 and 50% to A. nidulans NsdD, respectively. After 4 days of constant darkness cultivation, *CcnsdD2*, but not *CcnsdD1*, was upregulated on the first day of light/dark cultivation to induce fruiting bodies, and overexpression of *CcnsdD2*, but not *CcnsdD1*, produced more fruiting bodies under a light/dark rhythm. Although single knockdown of *CcnsdD2* did not affect fruiting body production due to upregulation of its homolog *CcnsdD1*, the double-knockdown CcNsdD1/NsdD2-RNAi transformant showed defects in fruiting body formation under a light/dark rhythm. Knockdown of *CcnsdD1*/*nsdD2* led to the differentiation of primary hyphal knots into sclerotia rather than secondary hyphal knots under a light/dark rhythm, similar to the differentiation of primary hyphal knots into sclerotia of the wild-type strain under darkness. The CcNsdD2-overexpressing transformant produced more primary hyphal knots, secondary hyphal knots, and fruiting bodies under a light/dark rhythm but only more primary hyphal knots and sclerotia under darkness. RNA-seq revealed that some genes reported previously to be involved in formation of hyphal knots and primordia, cyclopropane-fatty-acyl-phospholipid synthases *cfs1-3*, galectins *cgl1-3*, and hydrophobins *hyd1-3* were downregulated in the CcNsdD1/NsdD2-RNAi transformant compared to the mock transformant. ChIP-seq and electrophoretic mobility shift assay demonstrated that CcNsdD2 bound to promoter regulatory sequences containing a GATC motif in *cfs1*, *cfs2*, *cgl1*, and *hyd1*. A molecular mechanism by which CcNsdD2 regulates the developmental fate of *C. cinerea* under dark or light conditions is proposed.

## INTRODUCTION

The development of the basidiomycete fruiting body is a highly complex process that requires coordination between genetic, environmental, and physiological factors (1). In the model mushroom Coprinopsis cinerea, upon nutritional depletion, a single hypha locally undergo intense branching to form a small primary hyphal knot (also specifically termed hyphal knot) in the dark (1, 2). When continuously kept in the dark, the primary hyphal knot develops into sclerotium, a multicellular round brown-colored resting body (3). Under a “12 h light/12 h dark” rhythm, upon further branching, swelling, aggregation and fusion of hyphae, the primary hyphal knot develops to an enlarged secondary hyphal knot (also termed fruiting body initial) (1, 2). The secondary hyphal knot differentiates into a small fruiting body primordium with distinct cap and stipe tissues. Primordium development progresses continuously over days, resulting in distinct primordial stages, P1, P2, etc., up to P5, depending on cultivation time of 1 to 5 days after the occurrence of secondary hyphal knots (2). The primordium further develops into an immature fruiting body of approximately 15 to 20 mm in size. As the primordium gradually enlarges and matures, karyogamy and meiosis occur successively in basidia in the hymenium of the gills (1, 4), the stipe elongates, and the cap expands, giving rise to a mature fruiting body. Finally, basidiospores are formed, and the mature pileus autolyzes to release the basidiospores (1, 5, 6). If light is missing, the secondary hyphal knot will develop a “dark stipe” with an undeveloped cap and a slender stipe-like structure (1, 2, 7).

The putative blue light receptor proteins, WC-1/Dst1 (8), as well as its partner WC-2 (9), and Dst2 (10), were reported to be involved in fruiting body development in *C. cinerea*, and the *dst1*, *dst2*, or *wc-2* single mutants and a *dst1/dst2* double mutant produced a similar blind phenotype, forming the “dark stipe” (9, 10). Some genes, such as those encoding adaptor protein Cc.Ubc2 of the pheromone-responsive mitogen-activated protein (MAP) kinase cascade (11), chromatin remodeling regulator Cc.Snf5 (12), and arginine methyltransferase Cc. Rmt1 (13), were examined for their part in the hyphal cell clamp connection and primary hyphal knot formation in *C. cinerea* by genes rescuing defective phenotypes in mutants. Transcriptome analysis or expression pattern analysis revealed that some genes encoding cyclopropane-fatty-acyl-phospholipid synthases (*cfs*), galectins (*cgl*), *S*-adenosylmethionine-dependent methyltransferases (*ich*), hydrophobins (*hyd*), and laccases (*lcc*) were specifically upregulated during the transition from mycelium growth to initiation of primordia in *C. cinerea* (4, 6, 14–16). A cyclopropane-fatty-acyl-phospholipid synthase coding gene, *cfs1*, complemented the defect of fruiting body initiation in the AmutBmut UV mutant 6-031 of *C. cinerea* (5). The alteration of the physical properties of cellular membranes caused by *cfs1*-mediated production of cyclopropane-fatty-acyl-phospholipids may trigger fruiting body morphogenesis. In *C. cinerea*, galectin coding gene *cgl2* expression initiates in the dark at the time of primary hyphal knot formation, while *cgl1* expression initiates at the light-induced stage of secondary hyphal knot formation (4, 14). The mutant strains that formed primary hyphal knots but were unable to form fruiting body primordia were deficient in the expression of *cgl1* (4). Galectins were proposed to be involved in hyphal aggregation in the hyphal knot and primordium (16). A hydrophobin gene, *hyd9*, was reported to function in formation of the aerial hyphal knots and primordium in *Flammulina filiformis* (17). The *hyd9*-silenced transformant produced fewer primordia and fruiting bodies, whereas the *hyd9* overexpression transformant produced more primordia. Hydrophobins were proposed to reduce the tension between hyphae and the surface of the medium water to allow the mycelia to cross the water-air boundary into the air to form compact aerial hyphal knots and primordia (18, 19). *In vivo* and *in vitro* experiments showed that some chitinases and glucanases play important roles in stipe elongation growth (20–22) and pileus expansion and autolysis (23) during maturation of the fruiting body.

A central question in the study of genes involved in the morphogenesis of basidiomycete fruiting bodies is how their transcription is regulated by transcription factors to function in the specific developmental stage. The transcription factor *priB* was more abundantly expressed in the primordia of *Lentinus edodes* than in the preprimordial mycelia (24). A velvet transcription factor, two velvet-regulated transcript factors NsdD, and a homeodomain transcription factor STE-12 were shown to be upregulated in stage 1 primordia of 1 to 2 mm of *C. cinerea* grown under a 12-h light/dark rhythm for 3 days after 4 days of constant dark cultivation compared to vegetative mycelium grown only in the dark for 4 days (25). The transcription factors Fst3, Fst4, Hom1, Hom2, Bri1, C2h2, and Gat1 were found to inhibit or induce fruiting body development in *Schizophyllum commune* (26, 27). However, little information on the expression regulation of their target genes by these transcription factors is known during morphogenesis of basidiomycete fruiting bodies. In the ascomycete Aspergillus nidulans, some transcription factors, such as RlmA (28), BrlA (29–32), and NsdD (33–37), were found to be involved in sexual and asexual development, as well as the induction of some chitinases and β-1,3-glucanases.

We previously found that chitinases and β-1,3-glucanase cooperate together to involve in stipe elongation growth (20–22); therefore, in the preliminary experiment, we attempted to determine whether the homologs of some fungal transcription factors in *C. cinerea* play a role in stipe elongation growth through the regulation of chitinases and β-1,3-glucanases. Unexpectedly, we found that knockdown of the A. nidulans transcription factor NsdD homologs *CcnsdD2*/*nsdD1* led to a defect in light-induced formation of the primordium and fruiting body and that overexpression of *CcnsdD2* resulted in the formation of more primordia and fruiting bodies under light/dark rhythm conditions. Further studies explored the molecular mechanism by which CcNsdD2 regulates the developmental fate of *C. cinerea* under dark or light conditions.

## RESULTS

### Double knockdown of *CcnsdD1* and *CcnsdD2* resulted in impaired mycelial growth and defects in the production of fruiting bodies.

Protein BLAST analysis in NCBI using the GATA-type transcription factor NsdD (AAB16914.1) involved in the regulation of putative β-1,3-endoglucanase *eglC* in A. nidulans (33, 34) as a query revealed CC1G_12230, CC1G_14145, CC1G_14734, CC1G_01569, and CC1G_01461 from the *C. cinerea* okayama7#130 genome as putative homologs in addition to previously reported homologs CC1G_06265 and CC1G_06391 (25). Of these, CC1G_06265 (named CcNsdD1) and CC1G_12230 (named CcNsdD2) have sequence identities of 62 and 50% to A. nidulans NsdD, respectively, whereas the other five putative CcNsdD homologs show less than 50% identity to A. nidulans NsdD. CcNsdD1 and CcNsdD2 consist of 1117 and 700 amino acids (aa), respectively. Sequence alignment (see Fig. S1A) revealed that both CcNsdD1 and CcNsdD2 contain a nuclear localization signal (NLS) (38) and a conserved C-X_2_-C-X_18_-C-X_2_-C motif at the C terminus in all type IVb zinc-finger proteins with specificity for GATA motifs (39), whereas the other five putative CcNsdD homologs lack the nuclear localization signal. Phylogenetic analysis revealed that CcNsdD1 clustered as a group with basidiomycete *Laccaria bicolor* NsdD and *Schizophyllum commune* NsdD, which was phylogenetically close to those of most ascomycete species, while CcNsdD2 diverged from CcNsdD1 and clustered as a group only with Saccharomyces cerevisiae Gat2. However, other five putative *C. cinerea* NsdDs were distantly separated from CcNsdD1 and CcNsdD2, as well as other NsdD homologs of either ascomycetes or basidiomycetes (see Fig. S1B). There was no synteny in the chromosome location of CcNsdD1 and CcNsdD2 between three basidiomycetes: *C. cinerea*, *L. bicolor*, and *S. commune* (40). Identity analysis shows that CcNsdD1 and CcNsdD2 share only 15.22% of identify at the whole amino acid sequence. In addition, CcNsdD1 shares 66.67% of the amino acid sequence identity with CcNsdD2 in their DNA-binding domain, while 94.44% with *Laccaria bicolor* NsdD and *Schizophyllum commune* NsdD.

10.1128/mbio.03626-21.3 FIG S1 (A) Sequence alignment of NsdD homologs from different fungi of the ascomycetes and basidiomycetes. The nuclear localization sequence (NLS) was predicted by the NLStradamus (http://www.moseslab.csb.utoronto.ca/NLStradamus/) and marked in red (A. N. Nguyen Ba, et al., BMC Bioinformatics 10:202, 2009 [10.1186/1471-2105-10-202]. [19563654]). The GATA zinc finger domain was predicted by the NCBI Conserved Domain Search (https://www.ncbi.nlm.nih.gov/Structure/cdd/wrpsb.cgi?) and marked in blue (S. Lu, et al., Nucleic Acids Res 48:265–268, 2020 [10.1093/nar/gkz991] [31777944]). (B) Phylogenetic analysis of NsdD homologs from different fungi of the ascomycetes and basidiomycetes. The neighbor-joining (1,000 bootstraps) phylogenetic tree was constructed using MEGA 5 after ClustalW alignment. The scale bar indicates the genetic distance, which is proportional to the number of amino acid substitutions. Accession numbers of protein sequences in NCBI: *C. cinerea* NsdD1, CC1G_06265; *C. cinerea* NsdD2, CC1G_12230; *C. cinerea* NsdD3, CC1G_14145; *C. cinerea* NsdD4, CC1G_14734; *C. cinerea* NsdD5, CC1G_01569; *C. cinerea* NsdD6, CC1G_01461; *C. cinerea* NsdD7, CC1G_06391; *Laccaria bicolor* L297200, XP_001880936.1 (D. F. Plaza, et al., BMC Genomics 15:492, 2014 [10.1186/1471-2164-15-492] [24942908]); *Schizophyllum commune* S108660, XP_003032012.1 (D. F. Plaza, et al., BMC Genomics 15:492, 2014 [10.1186/1471-2164-15-492] [24942908]); Aspergillus fumigatus NsdD, Afu3g13870 (E. Szewczyk and S. Krappmann S, Eukaryot Cell 9:774–783, 2010 [10.1128/EC.00375-09] [20348388]); Aspergillus nidulans NsdD, AAB16914.1 (K. H. Han, et al., Mol Microbiol 41:299–309, 2001 [10.1046/j.1365-2958.2001.02472.x] [11489119]); Botrytis cinerea Ltf1, XP_024553024.1 (J. Schumacher, et al., PLoS Genet 10:e1004040, 2014 [10.1371/journal.pgen.1004040] [24415947]); Fusarium *fujikuroi* Csm1, XP_023430668.1 (E. M. Niehaus, et al., Front Microbiol 8:1175, 2017 [10.3389/fmicb.2017.01175] [28694801]); Metarhizium *rileyi* NsdD, QKU37907.1 (C. Xin, et al., Microb Biotechnol 13:1489–1501, 2020 [10.1111/1751-7915.13581] [32395911]); Neurospora crassa Sub-1, XP_011394474.1 (C. H. Chen, et al., EMBO J 28:1029–1042, 2009 [10.1038/emboj.2009.54] [19262566]); *Penicillium oxalicum*, EPS27088.1 (Q. P. He, et al., Appl Environ Microbiol 84:e01039-18, 2018 [10.1128/AEM.01039-18]); Sclerotinia sclerotiorum SsNsd1, ANQ80447.1 (J. Li, et al., Mol Plant Pathol 19:1679–1689, 2018 [10.1111/mpp.12651] [29227022]); Saccharomyces cerevisiae Gat2, DAA10033.1 (H. Forsberg, et al., Mol Microbiol 42:215–228, 2001 [10.1046/j.1365-2958.2001.02627.x] [11679080]). Download FIG S1, TIF file, 1.3 MB. Copyright © 2022 Liu et al. 2022 Liu et al. https://creativecommons.org/licenses/by/4.0/ This content is distributed under the terms of the Creative Commons Attribution 4.0 International license.

**FIG 1:**
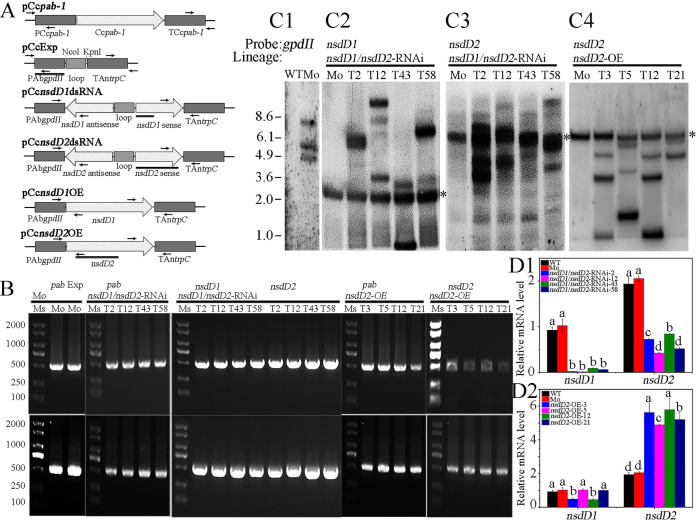
(A) Schematic representation of plasmids pCc*pab-1*, pCcExp, pCc*nsdD1*dsRNA, pCc*nsdD2*dsRNA, pCc*nsdD1*OE, and pCc*nsdD2*OE. The arrows and lines below the plasmids indicate the primers for genomic PCR and the hybridization probes for Southern blotting, respectively. (B) Genomic PCR shows that PCc*pab-1* (up) and TCc*pab-1* (down) from pCc*pab-1* and PAb*gpdII* (up) and TAn*trpC* (down) from pCcExp in the mock transformant, PCc*pab-1* (up) and TCc*pab-1* (down) from pCc*pab-1*, PAb*gpdII*-*nsdD1*antisense (up) and *nsdD1*sense-TAn*trpC* (down) from pCc*nsdD1*dsRNA, and PAb*gpdII*-*nsdD2*antisense (up) and *nsdD2*sense-TAn*trpC* (down) from pCc*nsdD2*dsRNA in the representative CcNsdD1/NsdD2-RNAi transformants (T2, T12, T43, and T58), PCc*pab-1* (up) and TCc*pab-1* (down) from pCc*pab-1*, and PAb*gpdII*-*nsdD2* (up) and *nsdD2*-TAn*trpC* (down) from pCc*nsdD2*OE in the representative CcNsdD2-OE transformants (T3, T5, T12, and T21), were integrated into the genome. The DL2000 DNA Marker (TaKaRa) was used as a size standard (Ms). (C) Southern blotting shows that the PAb*gpdII* of pCcExp in the mock transformants (Mo), the *nsdD1*sense of pCc*nsdD1*dsRNA and the *nsdD2*sense of pCc*nsdD2*dsRNA in the representative CcNsdD1/NsdD2-RNAi transformants (T2, T12, T43, and T58), and the *nsdD2* of pCc*nsdD2*OE in the representative CcNsdD2-OE transformants (T3, T5, T12, and T21) were integrated into the genome. Asterisks indicate the bands corresponding to the endogenous gene locus in the mock transformants. (D) qRT-PCR analysis of the relative transcript levels of *CcnsdD1* and *CcnsdD2* in the mycelia from four representatives of the CcNsdD1/NsdD2-RNAi transformants (T2, T12, T43, T58) and the CcNsdD2-OE transformants (T3, T5, T12, and T21) compared to wild-type AmutBmut (WT) and the mock transformants (Mo). The data are presented as the mean and standard error of three biological replicates (*n* = 9). A β-tubulin gene was used to standardize the mRNA level. The same letters indicate no significant difference (*P* > 0.05), and different letters indicate significant differences (*P* < 0.05) as determined by the Duncan test.

To determine *in vivo* function of *C. cinerea* CcNsdDs in fruiting body development, a hairpin double-stranded RNA (dsRNA)-mediated gene silencing strategy was used to downregulate the expression of *CcnsdD1* and/or *CcnsdD2* (20). The *C. cinerea* homothallic strain AmutBmut (*pab1-1*), which is fully self-compatible and can initiate fruiting body development under certain environmental conditions (1), was used as a recipient strain. Plasmid pCc*nsdD1*dsRNA containing the hairpin dsRNA of *CcnsdD1* (the alternative accession in NCBI: KAG2018048 in *C. cinerea* AmutBmut [41]) and plasmid pCc*nsdD2*dsRNA containing the hairpin dsRNA of *CcnsdD2* (the alternative accession in NCBI [KAG2011339] in *C. cinerea* AmutBmut [41]) were constructed and cotransformed along with pCc*pab-1* containing *pab1* as a selection marker into the haploid oidia to generate the single knockdown transformants CcNsdD1-RNAi and CcNsdD2-RNAi, respectively (Fig. 1A). Neither of the CcNsdD1-RNAi nor the CcNsdD2-RNAi transformants exhibited any apparent phenotype associated with fruiting body development under experimental conditions (see Fig. S2). Some lack of a phenotype in *CcnsdD1* or *CcnsdD2* single mutant may be due to the residual expression of the gene in knockdown strains, as shown by qRT-PCR (see Fig. S3B). However, qRT-PCR analysis explored that the expression of *CcnsdD2* was upregulated in the mycelia of the CcNsdD1-RNAi transformant (see Fig. S3B1) and that the expression of *CcnsdD1* was upregulated in the mycelia of the CcNsdD2-RNAi transformant (see Fig. S3B2) compared to that of the wild-type strain and the mock transformant, indicating that the function of silenced single *CcnsdD1* or *CcnsdD2* may be compensated by upregulating its homolog *CcnsdD2* or *CcnsdD1* (42, 43). Therefore, plasmids pCc*nsdD1*dsRNA, pCc*nsdD2*dsRNA and pCc*pab-1* were cotransformed into haploid oidia to generate the double-knockdown transformant CcNsdD1/NsdD2-RNAi. The empty plasmids pCcExp and pCc*pab-1* were cotransformed into haploid oidia to generate a mock transformant. Fifteen detected CcNsdD1/NsdD2-RNAi transformants or mock transformants were confirmed by genomic PCR (Fig. 1B) and Southern blotting (Fig. 1C1-3). qRT-PCR analysis showed that both *CcnsdD1* and *CcnsdD2* were strongly downregulated in all 15 different CcNsdD1/NsdD2-RNAi transformants (Fig. 1D1).

10.1128/mbio.03626-21.4 FIG S2 (A) Colonies (A1) of the representative wild-type parent AmutBmut strain (WT), mock transformant (Mo) and CcNsdD1-RNAi transformant (T1, T3, T5, and T9) after 4 days of the dark cultivation (4d D), and their fruiting bodies at 10:00 a.m. (A2) and at 14:00 p.m. (A3), respectively, on the sixth day of a 12 h light/12 h dark rhythm cultivation after 4 days of dark cultivation (4d D + 6d L/D). (B) Colonies (B1) of the representative wild-type parent AmutBmut strain (WT), mock transformant (Mo), and CcNsdD2-RNAi transformant (T1, T2, T4, and T9) after 4 days of dark cultivation (4d D), and their fruiting bodies at 10:00 a.m. (B2) and at 14:00 p.m. (B3), respectively, on the sixth day of a 12 h light/12 h dark rhythm cultivation after 4 days of dark cultivation (4d D + 6d L/D). Download FIG S2, TIF file, 3.0 MB. Copyright © 2022 Liu et al. 2022 Liu et al. https://creativecommons.org/licenses/by/4.0/ This content is distributed under the terms of the Creative Commons Attribution 4.0 International license.

10.1128/mbio.03626-21.5 FIG S3 (A) Genomic PCR shows that PAb*gpdII*-*nsdD1*antisense (up) and *nsdD1*sense-TAn*trpC* (down) from pCc*nsdD1*dsRNA (A1-2) in representative CcNsdD1-RNAi transformants (T1, T3, T5, and T9), PAb*gpdII*-*nsdD2*antisense (up) and *nsdD2*sense-TAn*trpC* (down) from pCc*nsdD2*dsRNA (A2-2) in representative CcNsdD2-RNAi transformants (T1, T2, T4, and T9), and PAb*gpdII*-*nsdD1* (up) and *nsdD1*-TAn*trpC* (down) from pCc*nsdD1*OE (A3-2) in representative CcNsdD1-OE transformants (T3, T4, T8, and T10), as well as PCc*pab-1* (up) and TCc*pab-1* (down) from pCc*pab-1* (A1-1, A2-1, and A3-1) in all transformants above, were integrated into the genome. The DL2000 DNA Marker was used as a size standard (Ms). (B) qRT-PCR analysis of the relative transcript levels of *CcnsdD1* and *CcnsdD2* in the mycelia from above four representatives of the CcNsdD1-RNAi, CcNsdD2-RNAi, and CcNsdD1-OE transformants, which were harvested after 4 days of dark cultivation compared to the wild-type parent AmutBmut strain (WT) and the mock transformant (Mo). The data are presented as the means and standard errors of three biological replicates (*n* = 9). A β-tubulin gene was used to standardize the mRNA level. The same letters indicate no significant difference (*P* > 0.05), and different letters indicate significant differences (*P* < 0.05) as determined by the Duncan test. Download FIG S3, TIF file, 0.7 MB. Copyright © 2022 Liu et al. 2022 Liu et al. https://creativecommons.org/licenses/by/4.0/ This content is distributed under the terms of the Creative Commons Attribution 4.0 International license.

**FIG 2:**
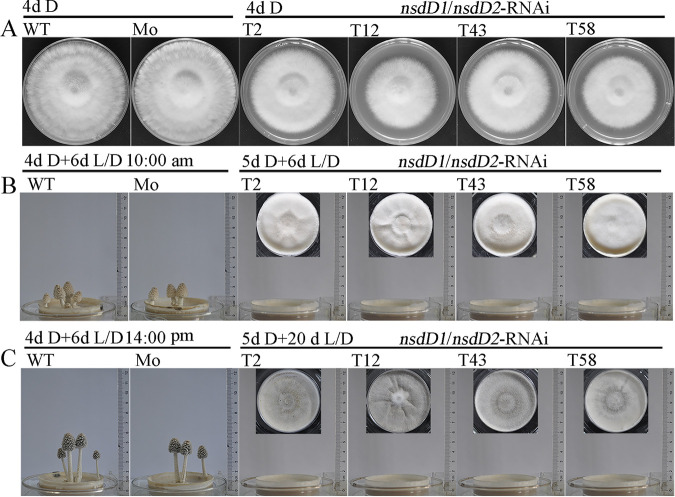
(A) Colonies of the representative wild-type parent AmutBmut strain (WT), mock transformant (Mo), and CcNsdD1/NsdD2-RNAi transformant (T2, T12, T43, and 58) after 4 days of cultivation in constant darkness (4d D). (B) Fruiting bodies of the representative wild-type parent AmutBmut strain (WT) and mock transformant (Mo) at 10:00 a.m. after 4 days of cultivation in continuous darkness followed by 6 days of cultivation under a 12 h light/12 h dark rhythm (4d D + 6d L/D), as well as colonies of the representative CcNsdD1/NsdD2-RNAi transformant after 5 days of cultivation in constant darkness, followed by 6 days of cultivation in a 12 h light/12 h dark rhythm (5d D + 6d L/D). (C) Fruiting bodies of the representative wild-type parent AmutBmut strain (WT) and mock transformant (Mo) at 14:00 pm after 4 days of cultivation in constant darkness followed by 6 days of cultivation under a 12 h light/12 h dark rhythm (4d D + 6d L/D), as well as colonies of the representative CcNsdD1/NsdD2-RNAi transformant after 5 days of cultivation in constant darkness, followed by 20 days of cultivation under a 12 h light/12 h dark rhythm (5d D + 20d L/D).

Unexpectedly, all 15 different detected double-knockdown CcNsdD1/NsdD2-RNAi transformants showed impaired mycelial growth and defects in fruiting body formation compared to the wild-type parent AmutBmut strain and the mock transformant. After 4 days of constant dark cultivation at 28°C, the mycelia of the wild-type parent AmutBmut strain or the mock transformant covered the entire agar medium surface, while the mycelia of the CcNsdD1/NsdD2-RNAi transformants were less developed and needed to be cultivated in the dark for an extra 1 day to cover the entire agar medium surface (Fig. 2A; see also Fig. S4A). When the mycelia of the wild-type AmutBmut strain or the mock transformant grown in constant darkness for 4 days until they covered the entire agar medium surface were placed under a 12 h light/12 h dark rhythm to continuously grow for an extra 6 days, they produced fruiting bodies. However, when the mycelia of the CcNsdD1/NsdD2-RNAi transformant grown in constant darkness for 5 days until they covered the entire agar medium surface were placed under the same light/dark rhythm to grow for an extra 6 days, they did not produce any fruiting bodies; even after 20 days of the light/dark rhythm incubation, they still did not form any fruiting bodies (Fig. 2B and C). As controls, the double-knockdown CcNsdD1/NsdD2-RNAi transformant was cultivated in constant darkness for 4 or 4.5 days and then transferred into the light/dark rhythm to continuously grow for an extra 6 days. It still did not produce any fruiting bodies, while the wild-type AmutBmut strain and mock transformant grown in constant darkness for 4.5 or 5 days, followed by 6 days of the light/dark rhythm cultivation, also still produced fruiting bodies (see Fig. S4).

10.1128/mbio.03626-21.6 FIG S4 The representative wild-type parent AmutBmut strain (WT), the mock transformant (Mo), and the CcNsdD1/NsdD2-RNAi transformant (T2, T12, T43, and T58) were cultivated in the constant darkness for 4 days, (4d D, A1), 4.5 days (4.5d D, A2), or 5 days (5d D, A3) and then transferred into a 12 h light/12 h dark rhythm to continuously grow for extra 6 days, indicated as 4d D + 6d L/D (B1), 4.5d D + 6d L/D (B2), and 5d D + 6d L/D (B3), respectively. Although the CcNsdD1/NsdD2-RNAi transformants showed different colony sizes depending on the time of the initial constant dark cultivation, they did not produce any fruiting body in following light/dark rhythm cultivation, whereas, although the mycelia of the wild-type AmutBmut strain and the mock transformant have covered the entire medium surface on day 4 of the initial constant darkness cultivation, the mycelia growing in the initial constant darkness for 4.5 or 5 days still produced fruiting bodies in the following light/dark rhythm cultivation. Download FIG S4, TIF file, 2.3 MB. Copyright © 2022 Liu et al. 2022 Liu et al. https://creativecommons.org/licenses/by/4.0/ This content is distributed under the terms of the Creative Commons Attribution 4.0 International license.

### Knockdown of *CcnsdD1*/*nsdD2* led to differentiation of primary hyphal knots into sclerotia rather than secondary hyphal knots under 12 h light/12 h dark rhythm conditions.

**FIG 3:**
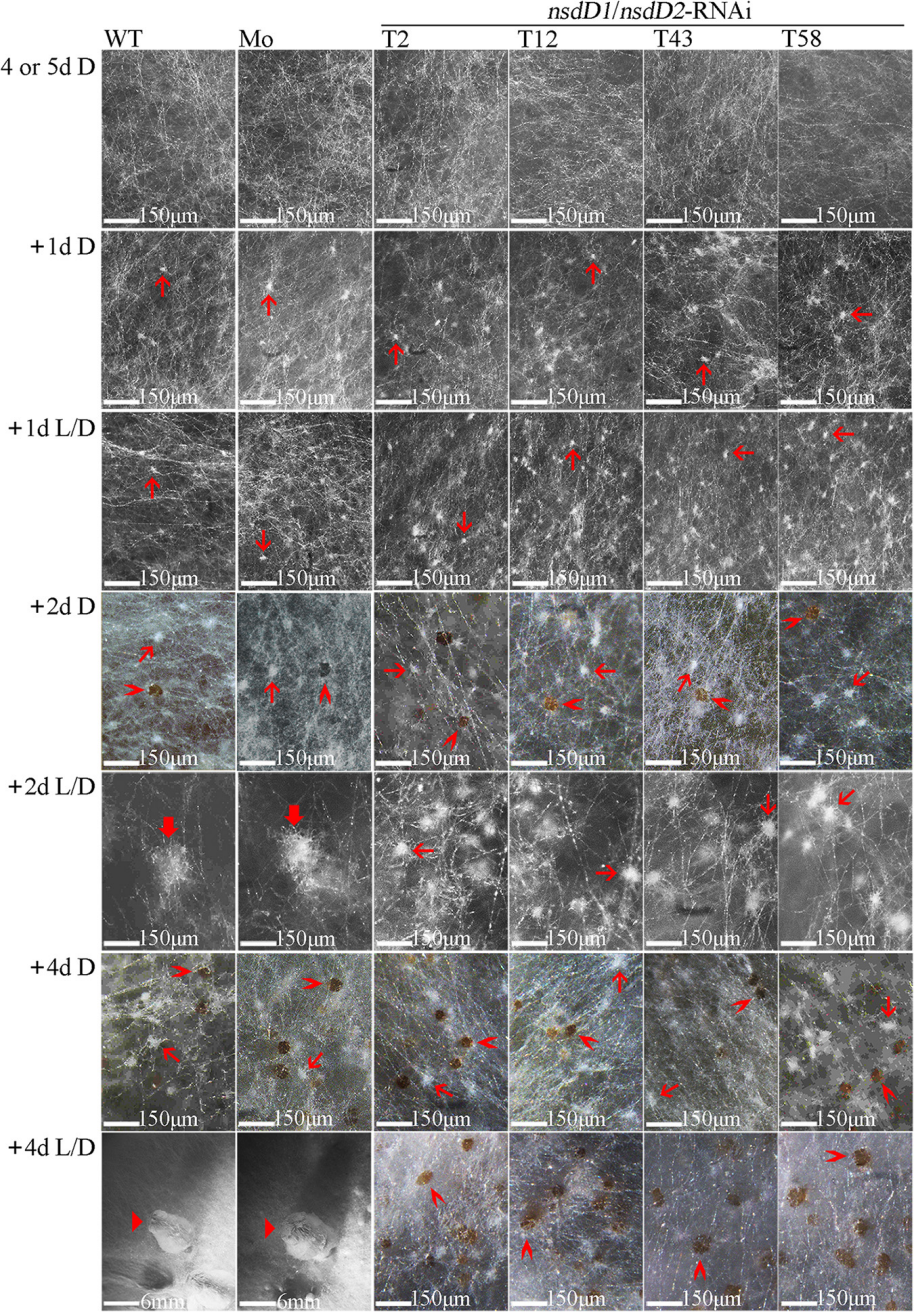
The development and differentiation of primary hyphal knots, sclerotia, secondary hyphal knots, and primordia from mycelia during cultivation of the representative wild-type parent AmutBmut strain (WT), mock transformant (Mo), and CcNsdD1/NsdD2-RNAi transformant (T2, T12, T43, and T58). Mycelia of *C. cinerea* were first cultivated in constant darkness (D) for 4 days (for AmutBmut strain and mock transformant) or 5 days (for CcNsdD1/NsdD2-RNAi transformant) until the mycelia covered the entire agar medium surface. Then, the mycelia were cultivated under a 12 h light/12 h dark rhythm (L/D) or under constant dark (D) conditions for 1 to 4 days. Thin red arrows, primary hyphal knot; thick red arrows, secondary hyphal knot; “**>**” red arrowheads, sclerotium; triangle (“▸”) red arrowheads, primordium. Scale bars are indicated.

After 4 days of dark cultivation (for the wild-type AmutBmut strain or the mock transformant) or 5 days of dark cultivation (for CcNsdD1/NsdD2-RNAi transformant) at 28°C until the mycelia covered the entire agar medium surface, none of the mycelia formed primary hyphal knots. Then, these mycelia were incubated at 28°C under a 12 h light/12 h dark rhythm or in constant darkness for the indicated days to compare their differentiation (Fig. 3). After 1 day of light/dark rhythm cultivation or dark cultivation, all mycelia in the wild-type strain, the mock transformant, and the CcNsdD1/NsdD2-RNAi transformant only formed similarly small primary hyphal knots (∼17 μm in diameter). After 2 days of light/dark rhythm cultivation, the primary hyphal knots of the wild-type AmutBmut strain and mock transformant differentiated into large secondary hyphal knots (∼150 μm in diameter), while the primary hyphal knots of the CcNsdD1/NsdD2-RNAi transformant did not differentiate into large secondary hyphal knots but still remained in a small primary hyphal knot status whose size only increased to ∼48 μm in diameter; in contrast, after 2 days of dark cultivation, all mycelia of the wild-type AmutBmut strain, the mock transformant, and the CcNsdD1/NsdD2-RNAi transformant produced only primary hyphal knots (∼25 μm in diameter) and brown sclerotia (∼32 μm in diameter). After 4 days of light/dark rhythm cultivation, the secondary hyphal knots of the wild-type AmutBmut strain and the mock transformant further developed to fruiting body primordia of ∼8 mm in height, while the primary hyphal knots of CcNsdD1/NsdD2-RNAi transformant only developed into brown sclerotia (approximately 60 μm in diameter); in contrast, after 4 days of dark cultivation, all wild-type AmutBmut strain, mock transformant and CcNsdD1/NsdD2-RNAi transformant only produced smaller primary hyphal knots (∼38 μm in diameter) and brown sclerotia (∼40 μm in diameter). These data indicated that knockdown of CcNsdD1/NsdD2 led to differentiation of primary hyphal knots into sclerotia rather than secondary hyphal knots under the light/dark rhythm, similar to the differentiation of primary hyphal knots into sclerotia of the wild-type parent AmutBmut strain or the mock transformant under the dark condition (1, 3).

### Knockdown of *CcnsdD* resulted in downregulation of some genes related to the formation of hyphal knots and primordia.

To characterize the transcription factor NsdD’s target genes controlling secondary hyphal knot formation in *C. cinerea*, mycelia of the CcNsdD1/NsdD2-RNAi transformant and the mock transformant grown at 28°C in constant darkness for 4 days (the colony size of the CcNsdD1/NsdD2-RNAi transformant was smaller than that of the mock transformant, as shown in Fig. 2A) were harvested to extract mRNA and construct a cDNA library for high-throughput Illumina sequencing. The differentially expressed genes (DEGs) between the CcNsdD1/NsdD2-RNAi transformant and mock transformant were determined using a threshold value of fold change >2 and an adjusted *P* value of <0.05. A total of 1,778 genes were identified as differentially expressed (see Data Set S1).

10.1128/mbio.03626-21.1 DATA SET S1 Global RNA-seq of the CcNsdD1/NsdD2-RNAi transformant and the mock transformant, the differentially expressed genes (DEGs; fold change > 2 and *P* < 0.05) between the CcNsdD1/NsdD2-RNAi transformant and the mock transformant in RNA-seq and the intersecting genes observed being differentially expressed in RNA-seq and identified as directly bound by NsdD2 in ChIP-seq, respectively. Download Data Set S1, XLSX file, 3.3 MB. Copyright © 2022 Liu et al. 2022 Liu et al. https://creativecommons.org/licenses/by/4.0/ This content is distributed under the terms of the Creative Commons Attribution 4.0 International license.

**FIG 4:**
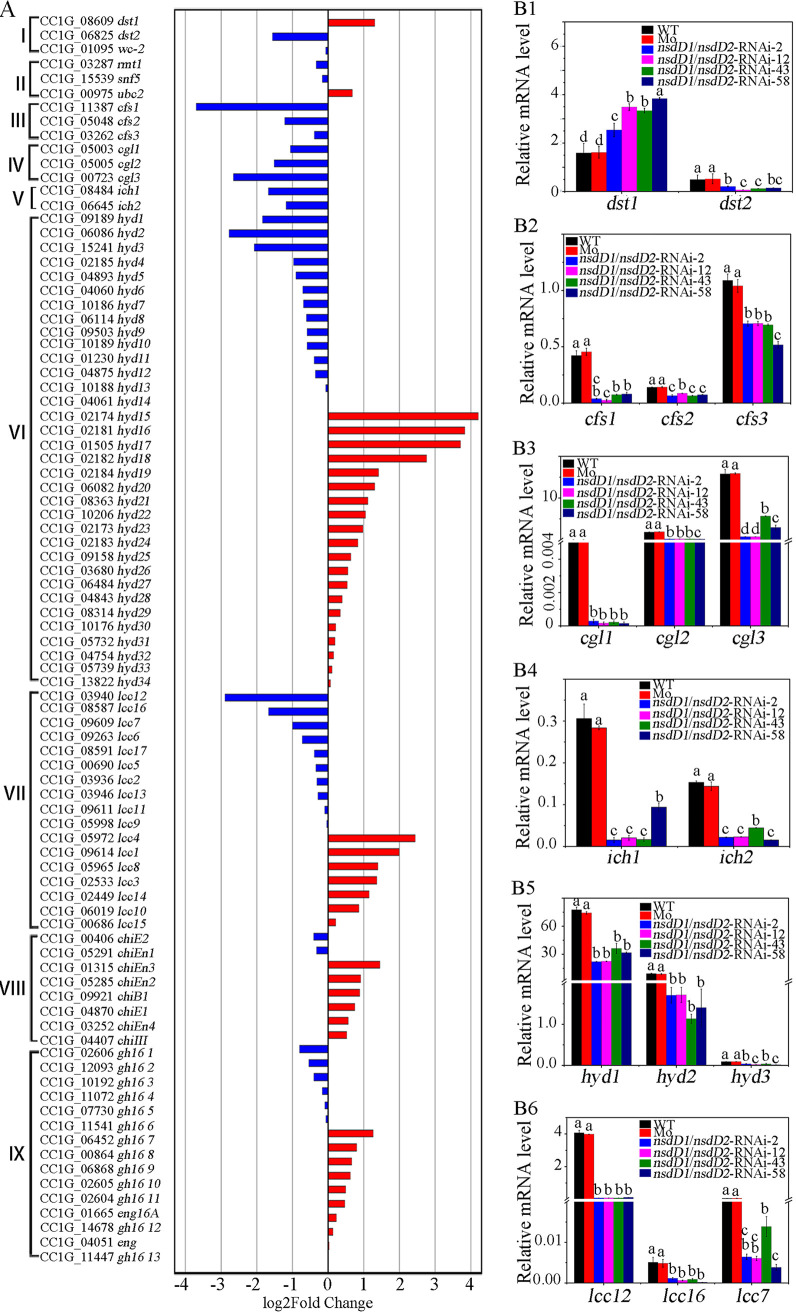
Double knockdown of *CcnsdD1*/*nsdD2* has a profound impact on expression of hyphal knot and primordium formation related genes. (A) RNA-seq explored partially selected genes with increased (red) and decreased (blue) expression in CcNsdD1/NsdD2-RNAi transformant relative to mock transformant. I, photoreceptor genes; II, cell clamp connection and primary hyphal knot formation-related genes; III, cyclopropane-fatty-acyl-phospholipid synthase genes (*cfs*); IV, galectin genes (*cgl*); V, *S*-adenosylmethionine-dependent methyltransferase (*ich*); VI, hydrophobin genes (*hyd*); VII, Laccases genes (*lcc*); VIII, chitinase genes, IX, GH16 family β-1,3-glucanase genes. (B) qRT-PCR analysis confirmed expression changes of some selected genes, *dst1-2* (B1), *cfs1-3* (B2), *cgl1-3* (B3), *ich1-2* (B4), *hyd1-3* (B5), *lcc* (*lcc12*, *lcc16*, and *lcc7*; B6) in the mycelia of representative CcNsdD1/NsdD2-RNAi transformant (T2, T12, T43, and T58) compared to the wild-type parent AmutBmut strain (WT) and the mock transformant (Mo). The data are presented as the means and standard errors of three biological replicates (*n* = 9). A β-tubulin gene was used to standardize the mRNA level. The same letters indicate no significant difference (*P* > 0.05), and different letters indicate significant differences (*P* < 0.05) as determined by the Duncan test.

RNA-seq showed that some genes reported previously to be involved in formation of hyphal knots and primordia were downregulated in the mycelia of the CcNsdD1/NsdD2-RNAi transformant compared to the mock transformant (Fig. 4A). For example, all three cyclopropane-fatty-acyl-phospholipid synthase genes (5, 16), *cfs1* (CC1G_11387), *cfs2* (CC1G_05048), and *cfs3* (CC1G_03262), were downregulated, although downregulation of *cfs3* showed a fold change <2 in the CcNsdD1/NsdD2-RNAi transformant compared to the mock transformant. All three galectin coding genes (4, 14), *cgl1* (CC1G_05003), *cgl2* (CC1G_05005), and *cgl3* (CC1G_00723), as well as two *S*-adenosylmethionine-dependent methyltransferase genes (16), *ich1* (CC1G_08484) and *ich2* (CC1G_06645), were downregulated in the CcNsdD1/NsdD2-RNAi transformant compared to the mock transformant. In addition, 3 of 34 hydrophobin genes (40), *hyd1* (CC1G_09189), *hyd2* (CC1G_06086), and *hyd3* (CC1G_15241), and 3 of 17 laccase genes (16, 44, 45), *lcc12* (CC1G_03940), *lcc16* (CC1G_09609), and *lcc7* (CC1G_08587), were downregulated in the CcNsdD1/NsdD2-RNAi transformant compared to the mock transformant. However, of reported photoreceptor protein genes (7), *dst1* (8) was upregulated, while *dst2* (10) was downregulated, and Dst1 partner WC-2 gene (9) was unchanged in the CcNsdD1/NsdD2-RNAi transformant compared to mock transformant. The expression of three genes reported to be involved in hyphal cell clamp connections and primary hyphal knot formation—*Cc.ubc2* (11), *Cc.snf5* (12), and *Cc.rmt1* (13)—did not show a significant difference between the CcNsdD1/NsdD2-RNAi transformant and the mock transformant because these genes are way upstream of hyphal knot formation. In addition, GH18 family chitinases (20) and GH16 family β-1,3-glucanases (21, 46, 47) related to stipe elongation were not significantly downregulated in the CcNsdD1/NsdD2-RNAi transformant compared to the mock transformant.

qRT-PCR analysis confirmed that *cfs1*, *cfs2*, and *cfs3* (Fig. 4B2); *cgl1*, *cgl2*, and *cgl3* (Fig. 4B3), *ich1* and *ich2* (Fig. 4B4); *hyd1*, *hyd2*, and *hyd3* (Fig. 4B5); and *lcc12*, *lcc16*, and *lcc7* (Fig. 4B6) were downregulated in the mycelia of the CcNsdD1/NsdD2-RNAi transformant compared to the mock transformant. Notably, although *dst1* and *dst2* were upregulated and downregulated, respectively, in the mycelia of the CcNsdD1/NsdD2-RNAi transformant (Fig. 4B1), *dst1* was downregulated in single knockdown of CcNsdD2-RNAi transformant compared to the mock transformant (see Fig. S6A2).

10.1128/mbio.03626-21.8 FIG S6 The qRT-PCR analysis of the relative transcript levels of *dst1* and *dst2* in the mycelia from four representatives of the CcNsdD1-RNAi (A1), CcNsdD2-RNAi (A2), CcNsdD1-OE (B1), and the CcNsdD2-OE (B2) transformants, which were harvested after 4 days of the constant dark cultivation compared to the wild-type parent AmutBmut strain (WT) and the mock transformant (Mo). The data are presented as the means and standard errors of three biological replicates (*n* = 9). A β-tubulin gene was used to standardize the mRNA level. The same letters indicate no significant difference (*P* > 0.05), and different letters indicate significant differences (*P* < 0.05) as determined by the Duncan test. Download FIG S6, TIF file, 0.2 MB. Copyright © 2022 Liu et al. 2022 Liu et al. https://creativecommons.org/licenses/by/4.0/ This content is distributed under the terms of the Creative Commons Attribution 4.0 International license.

### Some genes related to the formation of hyphal knots and primordia were identified as direct target genes of CcNsdD2.

We expressed the predicted C-terminal DNA-binding domains of CcNsdD1 (aa 565 to 869) and CcNsdD2 (aa 480 to 700) in Escherichia coli, designated CcNsdD1-C and CcNsdD2-C, respectively. Unfortunately, recombinant CcNsdD1-C was produced in the form of insoluble inclusion bodies in E. coli. After denaturation, purification, and refolding, the resulting soluble CcNsdD1-C was injected into New Zealand White rabbits, but the antibodies against CcNsdD1-C (anti-NsdD1-C) raised in the rabbits recognized only CcNsdD1-C but not CcNsdD1. Recombinant CcNsdD2-C was soluble and purified as an antigen for raising antibodies against CcNsdD2-C (anti-NsdD2-C) in New Zealand White rabbits, which recognized both CcNsdD2-C and CcNsdD2.

**FIG 5:**
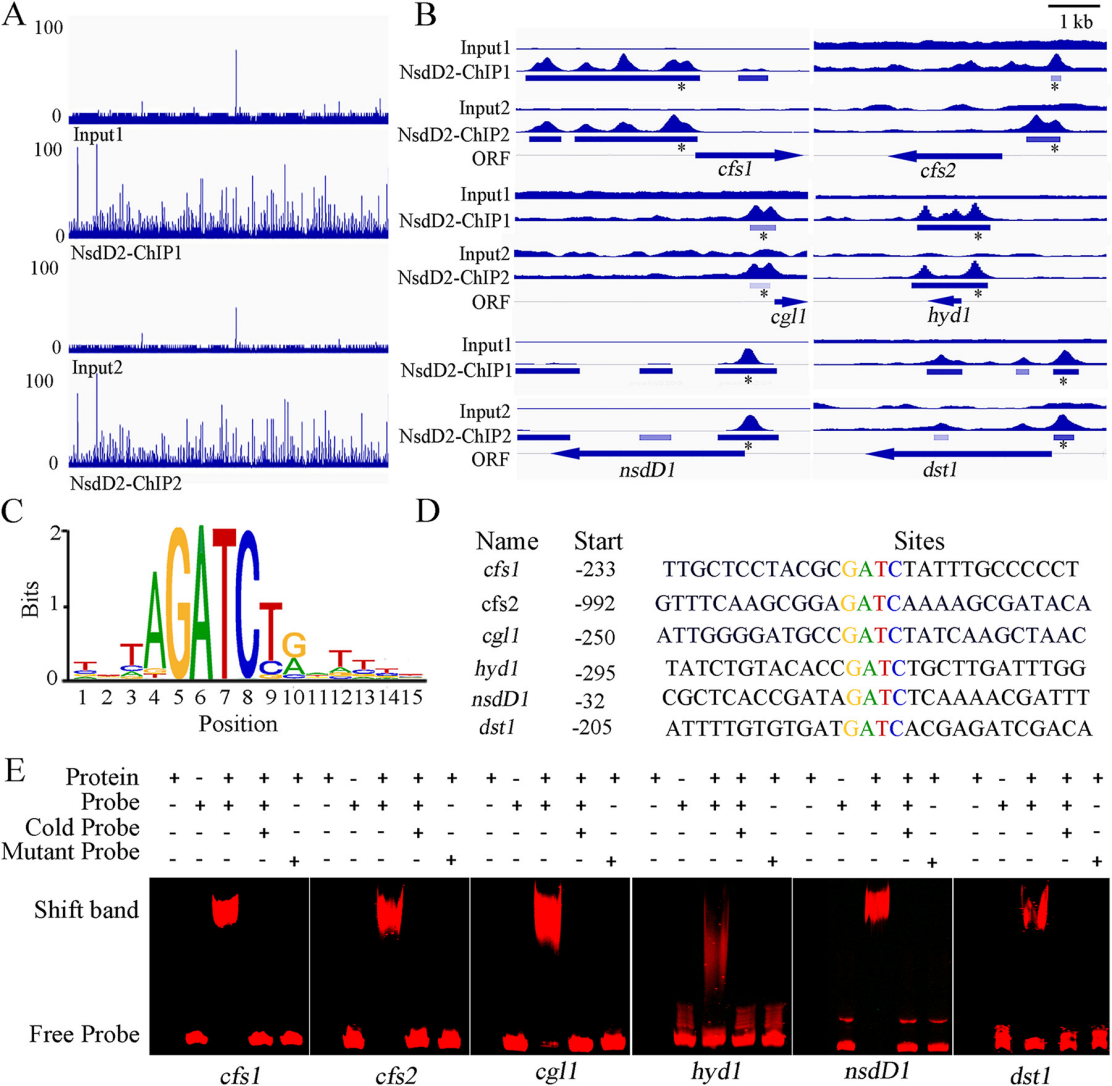
Some hyphal knot and primordium formation-related genes, the photoreceptor gene *dst1*, and NsdD2 paralogous gene *nsdD1* were identified as direct target genes of CcNsdD2 in the *C. cinerea* AmutBmut strain. (A) Genome-scale view of ChIP-seq data for the results of two independent CcNsdD2-C antibody ChIP samples (ChIP1 and ChIP2) and the relative input control samples. (B) ChIP-seq peaks for the hyphal knot and primordium formation-related genes *cfs1*, *cfs2*, *cgl1*, and *hyd1*, as well as photoreceptor *dst1* and paralog *nsdD1*, were among the highest ChIP-seq peaks and had relatively high enrichment versus the input control. Thick bars under peaks indicate the binding enrichment region from beginning to end in the chromatin. Asterisks indicate the location of NsdD2 binding motif in the binding enrichment region. Thick bar arrows indicate the open reading frame and the transcription direction of the indicated genes. (C) The CcNsdD2 binding motif was identified as GATC using MEME Suite analysis (E value = 1.3E−4). (D) GATC motifs in the CcNsdD2 binding sites of *cfs1*, *cfs2*, *cgl1*, *hyd1*, *dst1*, and *nsdD1* are shown. (E) EMSA of CcNsdD2 binding to the Cy5-labeled promoter fragments of *cfs1*, *cfs2*, *cgl1*, *hyd1*, *dst1*, and *nsdD1*. EMSA was performed by incubating the indicated amounts of purified CcNsdD2-C protein with a Cy5-labeled DNA fragment containing the GATC motif or a mutant ATAA motif instead of GATC. Competition was achieved by adding unlabeled probe (cold).

The anti-NsdD2-C antibody was used to perform chromatin immunoprecipitation followed by massively parallel DNA sequencing (ChIP-seq). Two independent ChIP-seq of the wild-type parent AmutBmut mycelia identified 8,994 peaks corresponding to 6,380 genes and 4,918 peaks corresponding to 4,064 genes (*q* < 0.05), respectively, sharing 4438 common peaks corresponding to 3741 common potential target genes (see Data Set S2). Of the 3,741 genes with associated NsdD2 DNA binding, the transcripts of 589 genes were identified as NsdD2 dependent by RNA-seq (see Data Set S1). Notably, both the ChIP-seq and RNA-seq gene set included several genes which were previously reported to be necessary for hyphal knot and primordium formation, *cfs1*, *cfs2*, *cgl1*, and *hyd1*. The peak intensity map of ChIP-seq (Fig. 5A and B: see also Data Set S2) shows that *C. cinerea* NsdD2 binding was significantly enriched at the region including intergenic region and promoter region (–3243 to + 63) of *cfs1*, the region from −524 bp upstream to + 958 bp downstream from the translational start site of *hyd1*, and the promoter regions of *cfs2* and *cgl1*. In addition, *C. cinerea* NsdD2 also bound to the promoter regions of photoreceptor gene *dst1* and NsdD2 homologous gene *nsdD1*. MEME analysis of 1-kb 5′-upstream regions of CcNsdD2-target genes from *C. cinerea* identified a highly conserved GATC motif localized approximately bp −992 to −32 upstream from the translation start site (Fig. 5C and D). In the putative CcNsdD2-target genes explored by ChIP-seq, *cfs1*, *cfs2*, *cgl1*, *hyd1*, *dst1*, and *nsdD1* all contained the GATC motif in their promoters.

10.1128/mbio.03626-21.2 DATA SET S2 Significant peaks and associated genes from two independent NsdD2 ChIP-seq analyses (*P* < 0.05) and common peaks and genes of two independent NsdD2 ChIP-seq analysis. Download Data Set S2, XLSX file, 1.2 MB. Copyright © 2022 Liu et al. 2022 Liu et al. https://creativecommons.org/licenses/by/4.0/ This content is distributed under the terms of the Creative Commons Attribution 4.0 International license.

Furthermore, electrophoretic mobility shift assay (EMSA) was used to detect the interaction between CcNsdD2 and promoter fragments of its putative target genes. As shown in Fig. 5E, CcNsdD2-C clearly interacted with Cy5-labeled promoter fragments (probe) with the GATC motifs: *cfs1*, *cfs2*, *cgl1*, *hyd1*, *dst1*, and *nsdD1*. Excess unlabeled DNA (cold probe) competitively blocked the interaction of CcNsdD2-C with these Cy5-labeled promoter fragments, and a Cy5-labeled probe containing a mutation of the GATC motif to ATAA in the promoter fragments (mutant probe) did not interact with CcNsdD2-C, underlining the specificity of the protein/DNA interaction. These findings supported the conclusion that these genes related to the formation of hyphal knots and primordia, *cfs1*, *cfs2*, *cgl1*, and *hyd1*, as well as photoreceptor *dst1* and the *nsdD2* paralog *nsdD1*, were the target genes of CcNsdD2.

### Light stimulation affected the expression of *CcnsdD2* and CcNsdD2-target hyphal knot formation-related genes and the developmental fate of *C. cinerea* primary hyphal knots after depletion of nutrients.

**FIG 6:**
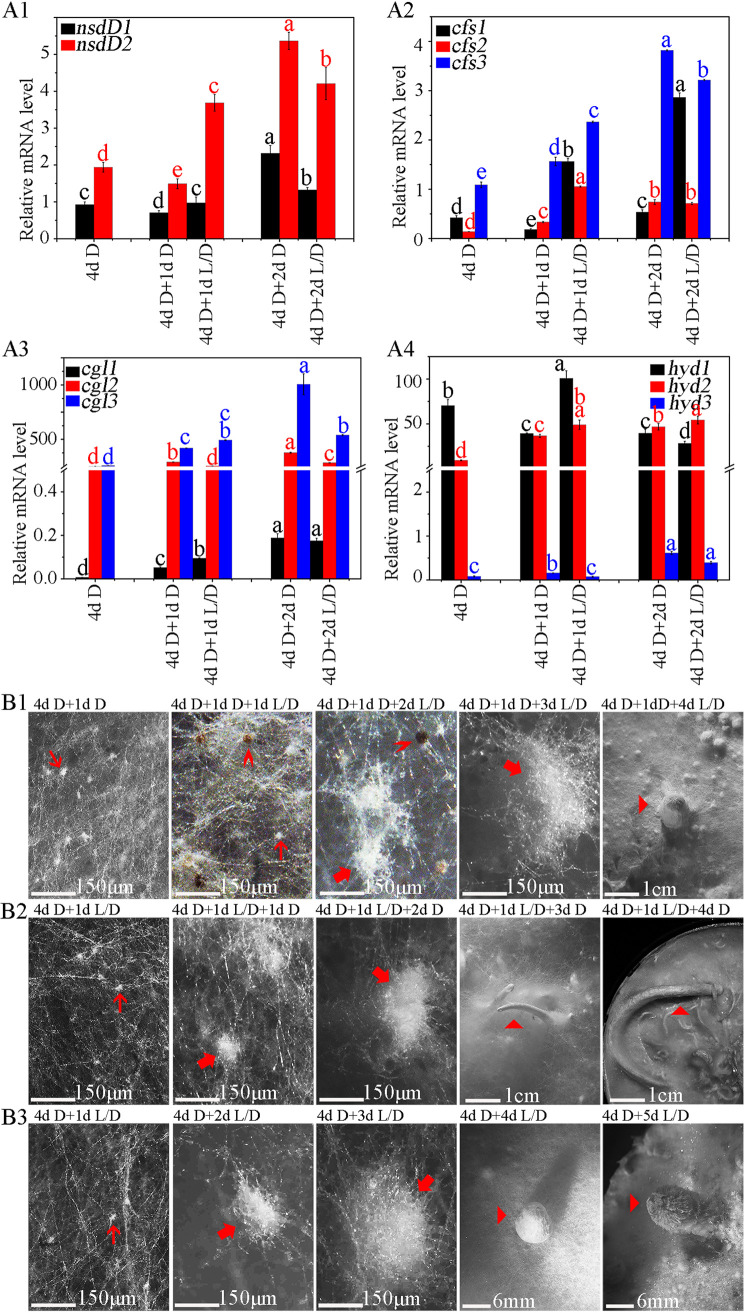
The effect of light stimulation or darkness after depletion of nutrients on the expression of *CcnsdD2* and CcNsdD2-target hyphal knot and primordium formation-related genes and the developmental fate of *C. cinerea* hyphal knots. (A) Expression of *CcnsdD1* and *CcnsdD2* (A1) and hyphal knot and primordium formation-related genes *cfs1-3* (A2), *cgl1-3* (A3), and *hyd1-3* (A4) in the wild-type AmutBmut strain mycelia grown in the dark for 4 days (4d D), grown continuously in darkness for 1 day or 2 days after 4 days of dark incubation (4d D + 1d D or 4d D + 2d D), or grown under a 12 h light/12 h dark rhythm for 1 day or 2 days after 4 days of dark incubation (4d D + 1d L/D or 4d D + 2d L/D). The data are presented as the means and standard errors of three biological replicates (*n* = 9). A β-tubulin gene was used to standardize the mRNA level. The same letters indicate no significant difference (*P* > 0.05), and different letters indicate significant differences (*P* < 0.05) as determined by the Duncan test. (B) After 4 days of dark cultivation until covering the entire agar medium surface, the mycelia of wild-type parent AmutBmut strain were cultivated in the darkness for an extra 1 day (4 d D + 1 d D) and then cultivated under 12 h light/12 h dark rhythm condition for 1 to 4 days (4 d D + 1 d D + 1–4 d L/D) (B1), or cultivated under 12 h light/12 h dark rhythm for only 1 day (4 d D + 1 d L/D) and then cultivated in the darkness for 1 to 4 days (4 d D + 1 d L/D + 1-4 d D) (B2), or cultivated under 12 h light/12 h dark rhythm for 1 to 5 days (4 d D + 1–5 d L/D) (B3) for control. Thin red arrows, primary hyphal knot; thick red arrows, secondary hyphal knot; “**>**” red arrowheads, sclerotium; triangle (“▸”) red arrowheads, primordium (B1 and B3) or dark stripe (B2). Scale bars are indicated.

The qRT-PCR analysis showed that the expression level of *CcnsdD2* increased by 90.20% and that the CcNsdD2-target genes—*cfs1*, *cfs2*, *cgl1*, and *hyd1*—were also correspondingly upregulated, while the expression level of *CcnsdD1* showed little change in the mycelia of wild-type AmutBmut strain grown under a 12 h light/12 h dark rhythm for an extra 1 day after 4 days of dark cultivation compared to mycelia grown in darkness for 4 days (Fig. 6A). In contrast, the expression level of *CcnsdD1* and *CcnsdD2* decreased by 23.71 and 23.10%, respectively, in the mycelia grown in the darkness for an extra 1 day after 4 days of dark cultivation compared to mycelia grown only in constant darkness for 4 days. Correspondingly, of the CcNsdD2-target genes, *cfs1* and *hyd1* were downregulated in the mycelia grown in the darkness for an extra 1 day after 4 days of dark cultivation compared to mycelia only grown in darkness for 4 days, Although the expression level of *cfs2* and *cgl1* in the mycelia grown in the darkness for an extra 1 day after 4 days of dark cultivation appeared higher than that in mycelia grown only in darkness for 4 days, their expression level was lower than that in mycelia grown in the light/dark rhythm for an extra 1 day. However, when the mycelia of wild-type AmutBmut strain were grown continuously in darkness for an extra 2 days after 4 days of dark cultivation, the expression levels of *CcnsdD1* and *CcnsdD2*, as well as CcNsdD2-target genes, *cfs1*, *cfs2*, and *cgl1*, but not *hyd1*, were also upregulated compared to the mycelia grown in the darkness for an extra 1 day after 4 days of dark cultivation, and some of the gene expression levels even were higher than that in the mycelia grown under light/dark rhythm for an extra 2 days after 4 days of dark cultivation. This indicates that only the first day of darkness or light/dark rhythm cultivation after 4 days of dark cultivation resulted in differential expression of *CcnsdD2* and its target genes in mycelia, although both the mycelia grown in darkness and mycelia grown under light/dark rhythm used for RNA extraction produced similar primary hyphal knots (Fig. 3).

We further determined effects of the first day of darkness or light/dark rhythm cultivation after 4 days of dark cultivation on the primary hyphal knot development. As shown in Fig. 6B1, after 4 days of dark cultivation, the wild-type AmutBmut mycelia were continuously incubated in the darkness for an extra 1 day and then grown under the light/dark rhythm for 1 to 4 days. They only formed primary hyphal knots and sclerotia on the first day of light/dark rhythm cultivation, similar to the mycelia grown in the dark for an extra 2 days after 4 days of dark cultivation (Fig. 3); however, they did not further produce more sclerotia but started to form some secondary hyphal knots on the second day of light/dark rhythm cultivation, and the latter further developed into fruiting body primordia after 4 days of light/dark rhythm cultivation. In contrast, after 4 days of darkness cultivation, if the culture was first grown under a light/dark rhythm for 1 day and then transferred to darkness to continuously grow for 1 to 4 days, wild-type AmutBmut mycelia still formed secondary hyphal knots, but the secondary hyphal knots finally developed dark stipes (Fig. 6B2), as described previously (1, 2, 7). This indicated that after 4 days of dark cultivation, upon depletion of nutrients, 1 day of dark or light/dark cultivation was vital in determining the developmental fate of *C. cinerea* hyphal knots.

### Overexpression of *CcnsdD2* promoted primary hyphal knot formation, secondary hyphal knot differentiation, and fruiting body production.

**FIG 7:**
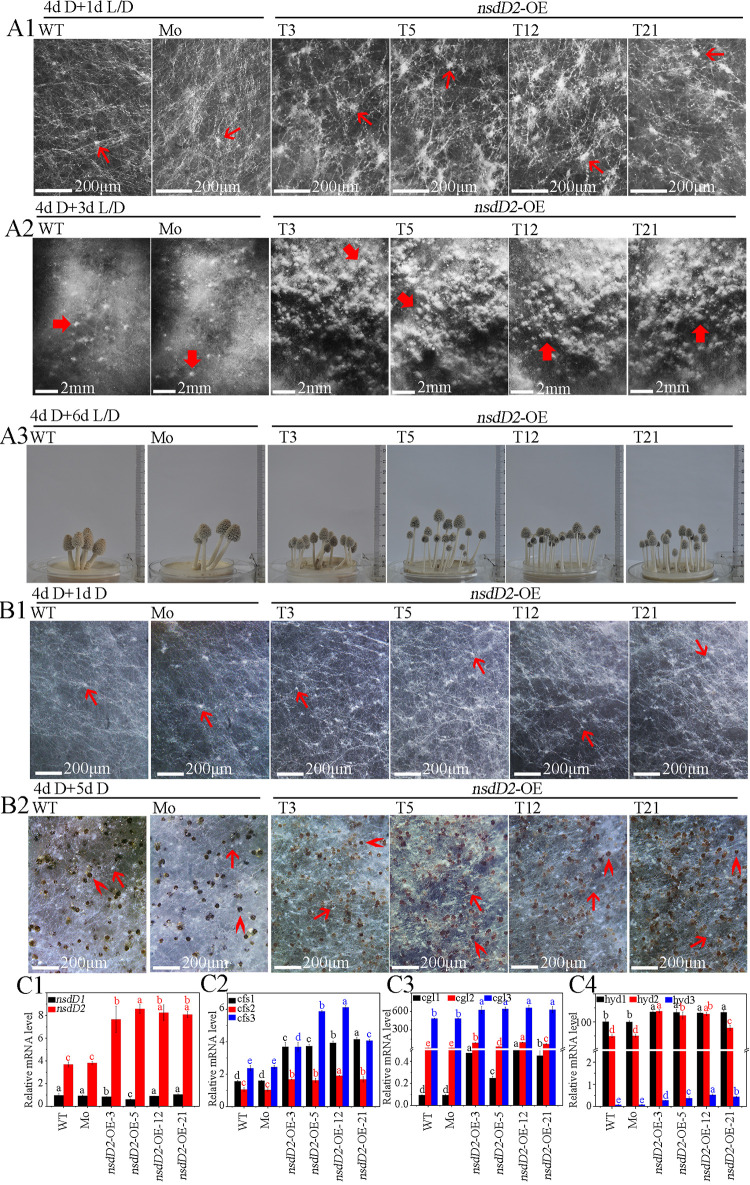
Effects of overexpressing *CcnsdD2* on the development and differentiation of primary hyphal knots, sclerotia, secondary hyphal knots, primordia, and fruiting bodies from mycelia during cultivation of *C. cinerea*. (A) Mycelia of the wild-type parent AmutBmut strain (WT), mock transformants (Mo), and representative CcNsdD2-OE transformant (T3, T5, T12, and T21) were first cultivated in the darkness for 4 days. Then, the mycelia were cultivated under a 12 h light/12 h dark rhythm for 1 day for observation of primary hyphal knots (4d D + 1d L/D) (A1), for 3 days for observation of secondary hyphal knots (4d D + 3d L/D) (A2), or for 6 days (4d D + 6d L/D) (A3) for observation of fruiting bodies. (B) After 4 days of dark cultivation, all mycelia were continuously cultivated in darkness for an extra 1 day for observation of primary hyphal knots (4d D + 1d D) (B1), or 5 days (4d D + 5d D) for observation of sclerotia (B2). Thin red arrows, primary hyphal knot; thick red arrows, secondary hyphal knot; “**>**” red arrowheads, sclerotium; triangle (“▸”) red arrowheads, primordium. Scale bars are indicated. (C) Expression of *CcnsdD1-2* (C1) and hyphal knot and primordium formation-related genes, *cfs1-3* (C2), *cgl1-3* (C3), and *hyd1-3* (C4) in mycelia of representative CcNsdD2-OE transformants (T3, T5, T12, and T21) relative to that of the wild AmutBmut parent strain (WT) or the mock transformant (Mo) grown in darkness for 4 days, followed by growth under a 12 h light/12 h dark rhythm for 1 day (4d D + 1d L/D). The data are presented as the means and standard errors of three biological replicates (*n* = 9). A β-tubulin gene was used to standardize the mRNA level. The same letters indicate no significant difference (*P* > 0.05), and different letters indicate significant differences (*P* < 0.05) as determined by the Duncan test.

To further determine the role of CcNsdD2 in fruiting, the expression plasmid p*CcnsdD2* was constructed (Fig. 1A) and transformed into the AmutBmut strain to generate an overexpressing *CcnsdD2* transformant, CcNsdD2-OE, in which *CcnsdD2* was overexpressed under the PA*bgpdII* promoter (the glyceraldehyde-3-phosphate dehydrogenase promoter from *Agaricus bisporus* [48]) (Fig. 1B, C4, and D2). Compared to the wild-type AmutBmut strain and the mock transformant, when mycelia of the CcNsdD2-OE transformant grown in darkness for 4 days until covering the entire medium surface were placed under a 12 h light/12 h dark rhythm to continuously grow for 1 day, they formed more primary hyphal knots (Fig. 7A1; see also Table S1), further produced more secondary hyphal knots after 3 days of light/dark rhythm incubation (Fig. 7A2; see also Table S1), and finally produced more fruiting bodies, increasing to 12.7 ± 2.8 plate^−1^ of fruiting bodies in CcNsdD2-OE from 4.5 ± 1.6 plate^−1^ of fruiting bodies in the mock transformant after 6 days of light/dark rhythm incubation (Fig. 7A3; see also Table S1). However, when mycelia of the CcNsdD2-OE transformant grown in darkness for 4 days were continuously grown in the darkness for another 5 days, they did not form secondary hyphal knots and primordia; instead, they only formed more primary hyphal knots on the first day (Fig. 7B1; see also Table S1) and finally produced more sclerotia on the fifth day (Fig. 7B2; see also Table S1) compared to the wild-type AmutBmut strain and mock transformant. In addition, the overexpressing *CcnsdD1* transformant (CcNsdD1-OE) did not show any apparent phenotype compared to the mock strain (see Fig. S5).

10.1128/mbio.03626-21.7 FIG S5 (A) Secondary hyphal knots from the mycelia of the representative the wild-type parent AmutBmut strain (WT), mock transformant (Mo), and CcNsdD1-OE transformant (T3, T4, T8, and T10). Mycelia of *C. cinerea* were first cultivated in constant darkness for 4 days until the mycelia covered the entire agar medium surface and then were cultivated under a 12 h light/12 h dark rhythm for 3 days (4d D + 3d L/D). (B) Fruiting bodies of the representative wild-type parent AmutBmut strain (WT), mock transformant (M), and CcNsdD1-OE transformant (T3, T4, T8, and T10) at 10:00 a.m. (B1) and at 14:00 p.m. (B2) on the sixth day of a 12 h light/12 h dark rhythm cultivation after 4 days of the dark cultivation (4d D + 6d L/D). Arrows indicate the secondary hyphal knot. Scale bars are shown. Download FIG S5, TIF file, 2.4 MB. Copyright © 2022 Liu et al. 2022 Liu et al. https://creativecommons.org/licenses/by/4.0/ This content is distributed under the terms of the Creative Commons Attribution 4.0 International license.

10.1128/mbio.03626-21.9 TABLE S1 Numbers of primary hyphal knots, secondary hyphal knots, fruiting bodies, and sclerotia produced by the wild-type AmutBmut strain, the mock transformant, and the CcNsdD2-OE transformant cultivated under the indicated conditions. Download Table S1, DOCX file, 0.02 MB. Copyright © 2022 Liu et al. 2022 Liu et al. https://creativecommons.org/licenses/by/4.0/ This content is distributed under the terms of the Creative Commons Attribution 4.0 International license.

The qRT-PCR results revealed that, compared to the wild-type AmutBmut strain and mock transformant, the CcNsdD2-OE transformant showed high expression of *CcnsdD2* and apparent upregulation of its target genes, *cfs1*, *cfs2*, *cgl1*, and *hyd1* (Fig. 7C), while downregulation of *dst1* (see Fig. S6B2). However, the CcNsdD1-OE transformant did not show upregulation of *CcnsdD2* (see Fig. S3B3).

## DISCUSSION

Although *CcnsdD1* was previously reported to be more highly expressed in the primordia than in the vegetative mycelia of *C. cinerea* (25), its role in the development of the fruiting body has not been elucidated. This study found that on the first day of light/dark rhythm cultivation after 4 days of dark cultivation, the *C. cinerea* wild-type AmutBmut strain produced primary hyphal knots with the capacity to develop secondary hyphal knots and primordia, but it did not upregulate *CcnsdD1*. Furthermore, knockdown or overexpression of *CcnsdD1* did not affect secondary hyphal knot formation or fruiting body production. However, *CcnsdD2*, was upregulated in the AmutBmut strain by the first day of light/dark rhythm cultivation after 4 days of dark cultivation, and the overexpressing *CcnsdD2* transformant produced more secondary hyphal knots, primordia, and fruiting bodies under the light/dark rhythm than the wild-type AmutBmut strain. Thus, there may be a functional diversification between paralogous *CcnsdD1* and *CcnsdD2* in addition to a functional redundancy, which is similar to the functional diversification, redundancy, and epistasis among paralogs of the Drosophila melanogaster *obp50a–d* gene cluster (49). Therefore, CcNsdD1 may not function in the regulation of fruiting body production in the case of normally expressing *CcnsdD2* which is dominant to *CcnsdD1*, which is consistent with the previous report that some genes were dominant to their loss-of function paralogous genes for human Mendelian diseases (50). Although the single knockdown of *CcnsdD2* did not affect fruiting, the double knockdown of *CcnsdD1* and *CcnsdD2* led to the absence of a secondary hyphal knot, primordium, and fruiting body. We presume that CcNsdD2 mainly functions in the formation of the hyphal knot and primordium. The lack of any apparent phenotype associated with fruiting body development in the single knockdown of *CcnsdD2* transformant may be due to the upregulation of *CcnsdD1* in the CcNsdD2-RNAi transformant (see Fig. S3B2), which may partially compensate for the function of silenced *CcnsdD2* (42, 43). Given facts that CcNsdD2 could bind to *CcnsdD1* promoter and overexpressing *CcnsdD2* transformant showed a trend of downregulation of *CcnsdD1* (Fig. 1D2), perhaps CcNsdD2 is a repressor for *CcnsdD1* expression and knockdown of CcNsdD2 allows repressed *CcnsdD1* to be upregulated (42, 43).

**FIG 8:**
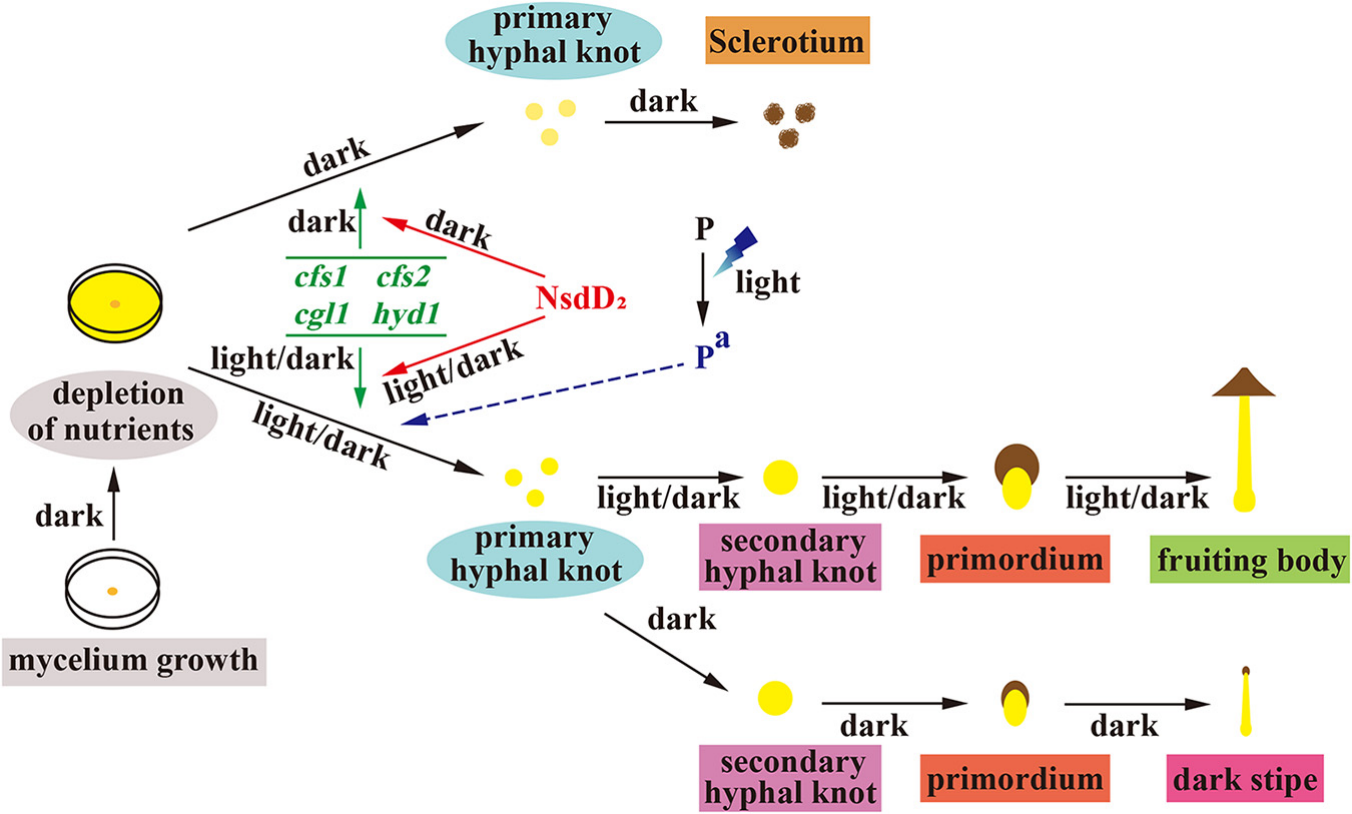
Working model for CcNsdD2 regulating the developmental fate of *C. cinerea* under darkness or 12 h light/12 h dark rhythm conditions. CcNsdD2 promotes the formation of primary hyphal knots by upregulating the genes *cfs1*, *cfs2*, *cgl1*, and *hyd1*. However, the development and fates of the primary hyphal knots depend on light stimulation after 4 days of dark cultivation until mycelia cover the entire agar medium surface. One day of 12 h light/12 h dark rhythm cultivation is presumed to result in activation of the putative photoreceptor protein P. Activated P^a^ controls the differentiation of primary hyphal knots into secondary hyphal knots together with CcNsdD2 after light stimulation. Either the downregulation of CcNsdD2, such as in the CcNsdD1/NsdD2-RNAi transformant under the light/dark rhythm, or the absence of light-activated P^a^, such as in the wild-type AmutBmut strain in the dark, results in the development of primary hyphal knots into sclerotia. In addition, the secondary hyphal knot formed under light/dark rhythm develops into a dark stipe under darkness. The interaction between NsdD2 and corresponding photoreceptors in the photomorphogenesis of primordium needs to be elucidated in the future. The color continuous lines are supported by experimental evidence, and the color dotted lines are presumed.

Some genes, such as cyclopropane-fatty-acyl-phospholipid synthase coding gene *cfs* (5), galectin coding gene *cgl* (4, 14, 16), and hydrophobin coding gene *hyd* (6, 17), were reported previously to be involved in hyphal knot and primordium formation of basidiomycetes. Consistent with the promotion of CcNsdD2 in hyphal knot and primordium formation, this study demonstrated that three *cfs1-3* (5), three *cgl1-3* (4), and three *hyd1-3* (40) were upregulated in the mycelia of wild-type parent AmutBmut strain grown under the light/dark rhythm for 1 day after 4 days of dark cultivation compared to that in mycelia grown in the dark for 4 days and were downregulated in double knockdown of CcNsdD1/NsdD2-RNAi transformant while upregulated in overexpressing *CcnsdD2* transformant compared to wild-type AmutBmut strain. Previously, *cfs1-3*, *cgl1-3*, and *hyd2* were shown to be upregulated in the mycelium with hyphal knots of *C. cinerea* after 1 day of light exposure compared to the mycelium grown in the dark before light exposure (51), and *cfs1* and *cfs2* were even highly expressed after 1 h of light exposure in the mycelial region where the hyphal knot would be developed (16). Together, these results indicate that CcNsdD2 promotes hyphal knot and primordium formation by upregulating some hyphal knot and primordium formation-related genes (Fig. 8).

ChIP-seq analysis further demonstrated that CcNsdD2 directly bound to the regulatory sequences of *cfs1*, *cfs2*, *cgl1*, and *hyd1 in vivo*. Using MEME analysis, the CcNsdD2 DNA binding motif was identified as a GATC motif. Although fungal GATA transcription factors such as A. nidulans NsdD usually bind to the GATA motif of the target gene promoter (36, 52), some fungal light-responsive GATA transcription factors such as Wc-1 and Sub-1 bind to GATC motif of their target gene promoters (53). The significance of interaction between light responsive transcription factors and GATC motif of the target gene promoter needs to be clarified. The GATC motif exists within the 1,000 bp upstream area of the promoters of the above putative target genes of CcNsdD2. EMSA analyses confirmed that CcNsdD2 bound to GATC motifs *in vitro* in the promoters of *cfs1*, *cfs2*, *cgl1*, and *hyd1* in *C. cinerea*. These results collectively indicate that CcNsdD2 is an activator that directly binds in the promoter regions of *cfs1*, *cfs2*, *cgl1*, and *hyd1* to positively regulate their expression for fruiting in *C. cinerea*. Therefore, *nsdD2* as likely to act upstream of hyphal knot initiation and *cfs1*, *cfs2*, *cgl1*, and *hyd1* may be the direct target genes of CcNsdD2 (Fig. 8). Other hyphal knot and primordium formation-related genes may be indirectly upregulated by CcNsdD2. In the ascomycete A. nidulans, NsdD was reported to positively regulate sexual reproduction (34). Deletion of *nsdD* resulted in no cleistothecia (fruiting bodies) formation, whereas overexpression of *nsdD* increased the number of cleistothecia (34). Our results provide new experimental evidences for convergent evolution and similarity between Ascomycota and Basidiomycota sexual fruiting bodies (54). However, no gene has been reported to be a direct target of NsdD for production of sexual cleistothecia in A. nidulans. Thus far, only one gene, *brlA*, was reported to be a direct target gene of NsdD and NsdD as a repressor repressed *brlA* expression and asexual conidiation in A. nidulans (36).

Morphogenetic analysis of the wild-type AmutBmut strain showed that the primary hyphal knots formed on the first day of light/dark rhythm cultivation after 4 days of dark cultivation developed into secondary hyphal knots on the following day of dark cultivation, whereas the primary hyphal knots formed on the first day of extra dark cultivation after 4 days of dark cultivation developed into sclerotia on the following day of light/dark rhythm cultivation. In addition, after 4 days of dark cultivation, the CcNsdD2-OE transformant grown either in the dark or under the light/dark rhythm for 1 day formed more primary hyphal knots than the wild-type AmutBmut strain and mock transformant, but the former developed into sclerotia under the light/dark rhythm and the latter into fruiting bodies in the dark. These results collectively indicate that although NsdD2 promotes primary hyphal knot formation, the developmental fates of the primary hyphal knots depend on whether light/dark cultivation or dark cultivation occurs after 4 days of dark cultivation. Interestingly, double knockdown of *CcnsdD1* and *CcnsdD2* did not apparently affect the formation of primary hyphal knots. Perhaps the CcNsdD1/NsdD2-RNAi transformant normally expresses hyphal cell clamp connections and primary hyphal knot formation-related genes (Fig. 4A)—*Cc.ubc2* (11), *Cc.snf5* (12), and *Cc.rmt1* (13)—as an alternative pathway to maintain primary hyphal knot formation.

However, after 4 days of dark cultivation until mycelia covered the entire agar medium surface, although the expression of *CcnsdD2* was upregulated in the mycelia of the wild-type AmutBmut strain on the first day of the following light/dark rhythm cultivation and downregulated on the first day of the following dark cultivation, *CcnsdD2* expression was also upregulated on the second day of the following dark cultivation. In addition, after 4 days of dark cultivation, although the CcNsdD2-overexpressing transformant produced more primary hyphal knots, secondary hyphal knots, and fruiting bodies under the light/dark rhythm, it only produced more primary hyphal knots and sclerotia under the dark and did not completely overcome the light requirement for fruiting. These results collectively indicated that the induction of *CcnsdD2* is not under direct control of light and photoreceptors. We presumed that *C. cinerea* has a putative light-activated photoreceptor protein that controls the differentiation of primary hyphal knots into secondary hyphal knots together with CcNsdD2 after light stimulation (Fig. 8). That is, the putative light-activated photoreceptor is needed before hyphal knots formation for destining the developing hyphal knots to the fruiting body lineage as opposed to the sclerotium lineage. Therefore, high expression of *CcnsdD2* alone did not lead to differentiation of primary hyphal knots into secondary hyphal knots in the dark because of the lack of the light-activated photoreceptor. In the same light, the double-knockdown transformant only formed sclerotia rather than secondary hyphal knots and primordia under the light/dark rhythm, which should be due to the expression level of *CcnsdD2* being lower than a critical concentration required for triggering secondary hyphal knot development and primordium formation, despite the presence of the putative light-activated photoreceptor protein. Apparently, the role of CcNsdD2 in sclerotium formation in *C. cinerea* is different from that reported in ascomycete species. In the plant-pathogenic ascomycete Sclerotinia sclerotiorum, deleting the A. nidulans NsdD ortholog SsNsd1 mutant produced smaller and lighter sclerotia with loss of cell integrity (55). In Botrytis cinerea (38) and A. flavus (56), deleting A. nidulans NsdD homolog mutants even failed to produce any sclerotium.

It is known that blue light stimulates the initiation of fruiting bodies in *C. cinerea* (7, 16, 57–59). The putative blue light receptors, Dst1 (WC-1) (8), and Dst2 (10), as well as Dst1 partner WC-2 (9), for fruiting body development have been identified in *C. cinerea*. The Dst1, WC-2, or Dst2 single mutant or the Dst1/Dst2 double mutant produced a blind phenotype which normally formed secondary hyphal knots and primordia, but primordia only developed dark stipes under a light/dark rhythm (7–10). However, double knockdown of CcNsdD1/NsdD2-RNAi transformant only produced sclerotia but not any secondary hyphal knot and primordium under a light/dark rhythm. Therefore, it is impossible for CcNsdD2 to function as a direct downstream target of the reported photoreceptors, Dst1 and Dst2. Furthermore, RNA-seq revealed that Dst1 was upregulated, Dst2 was downregulated, and Wc-2 was unchanged in the CcNsdD1/NsdD2-RNAi transformant compared to the mock transformant, and ChIP-seq showed that NsdD2 only bound to the *dst1* promoter but not *wc-2* and *dst2*. Therefore, *wc-2* and *dst2* are also not induced directly by NsdD2. The expression of *dst1* is even suppressed by NsdD2 because overexpression of CcNsdD2 downregulated *dst1* and double knockdown of CcNsdD1/NsdD2 upregulated *dst1*, while downregulation of *dst1* in a single knockdown of CcNsdD1/NsdD2-RNAi transformant may be due to the paralogous compensation of upregulated *CcnsdD1* (42, 43). Together, NsdD2 and above-reported photoreceptors do not function through inducing mutual gene expression in photomorphogenesis of primordium. This further supports our hypothesis by which some reported or unknown photoreceptor protein(s) may cooperate with NsdD2 to play a role in photomorphogenesis of primordium after being activated by light (Fig. 8). In the future, it is necessary to further elucidate the mechanism of interaction between NsdD2 and corresponding photoreceptors in the photomorphogenesis of primordium.

## MATERIALS AND METHODS

### Strains and cultures.

Coprinopsis cinerea strain AmutBmut (*A43mut B43mut pab1-1*) was purchased from the Japan Collection of Microorganisms (JCM; Japan).

For cultivation of mycelium, fruiting bodies and sclerotia, an agar block with mycelium of the AmutBmut strain or transformants of AmutBmut was inoculated on the center of PDYA medium agar in petri dishes 7 cm in diameter and incubated at 28°C in constant darkness in an incubator for 4 days until the mycelia covered the entire medium surface; then, the mycelia on the petri dishes were transferred at 28°C to a 12 h light/12 h dark rhythm or to constant darkness in the incubator to continuously grow for the indicated number of days, unless stated otherwise (60, 61). Constant darkness was provided by ventilated, light-proof boxes in the incubator, and light (40 to 50 μmol m^−2^ s^−1^) was provided by white fluorescent tubes in the incubator.

Photographs of mycelia, primary hyphal knots, sclerotia, secondary hyphal knots, primordia, dark stipes, and fruiting bodies were taken using a Nikon SMZ1500 stereoscopic zoom microscope with a Nikon HR Plan Apo 1× WD 54 objective and a Nikon digital sight DS-Fi2 system or a Nikon digital camera D-90 (62).

### Construction of plasmids and DNA transformation.

The expression plasmids pCc*pab-1* with *C. cinerea Ccpab1* promoter and terminator and pCcExp with an *Agaricus bisporus gpdII* promoter and an Aspergillus nidulans *trpC* terminator were constructed as described previously (20). For construction of gene silencing plasmids, nucleotide bp 750 to 1 (nucleotides were numbered from the predicted translational start site) of the antisense fragment sequence and bp 101 to 750 of the sense fragment sequence of *CcnsdD1* or *CcnsdD2* cDNA without any intron were ligated into the NocI and KpnI sites, respectively, of pCcExp to generate plasmids pCc*nsdD1*dsRNA and pCc*nsdD2*dsRNA, respectively; for construction of the gene-overexpressing plasmids, bp 1 to 3746 of the full-length sequence of the *CcnsdD1* gDNA fragment including introns or bp 1 to 2468 of the full-length sequence of the *CcnsdD2* gDNA fragment including introns were ligated into the NocI and KpnI sites, respectively, of pCcExp to generate plasmids pCc*nsdD1*-OE and pCc*nsdD2*-OE, after digestion with NocI and KpnI (20, 48, 63, 64).

For DNA transformation, protoplasts were prepared from the oidia of strain AmutBmut and cotransformed with pCc*pab-1* and the indicated gene silencing plasmids, gene-overexpressing plasmids or empty plasmids by polyethylene glycol/CaCl_2_ methods, as previously described (20, 65).

### Genomic PCR analysis.

Genomic DNAs prepared from the mycelia of the transformants using a UNlQ-10 column fungal genomic DNA isolation kit (Sangon) were used as templates to be amplified by PCR using two pairs of primers for each plasmid (see Table S2). One pair of primers amplified a fragment between the plasmid backbone and promoter, and one pair of primers amplified a fragment between the terminator and plasmid backbone to check the integration of pCcpab-1 or pCcExp in the genome; one pair of primers amplified a fragment between the PAb*gpdII* and inserted sequence 5′-terminal, and one pair of primers amplified a fragment between inserted sequence 3′-terminal and TAntrpC to check the integration of pCc*nsdD1*dsRNA, pCc*nsdD2*dsRNA, pCc*nsdD1*OE, and pCc*nsdD2*OE in the genome (20).

10.1128/mbio.03626-21.10 TABLE S2 Primers used in this study. Download Table S2, DOCX file, 0.03 MB. Copyright © 2022 Liu et al. 2022 Liu et al. https://creativecommons.org/licenses/by/4.0/ This content is distributed under the terms of the Creative Commons Attribution 4.0 International license.

### Southern blotting.

Genomic DNAs prepared by the cetyltrimethylammonium bromide (CTAB) method were digested with HindIII, separated on a 0.7% agarose gel, and transferred to a nylon membrane (Zeta-Probe+; Roche). The preparation of the hybridization probes using probe primers (see Table S2) and detection of the specific DNA fragments were performed as previously described (20).

### qRT-PCR analysis.

Total RNA extracted from the indicated mycelia or fruiting bodies using a spin column fungal total RNA purification kit (Sangon Biotech) was used to synthesize first-strand cDNA using a HiScript II Q RT Supermix for qPCR kit (+ gDNA wiper) (Vazyme), and the resulting cDNA was used for qRT-PCR analysis using a pair of specific primers for each gene (see Table S2) and AceQ qPCR SYBR green Master Mix (Vazyme) (20). The gene expression levels were normalized to β-tubulin, and the fold expression of target genes relative to β-tubulin was calculated according to the 2^−Δ^*^CT^* method as follows: −Δ*C_T_* = −(*C_T _*_target_ − *C_T _*_β-tubulin_).

### RNA-seq.

The CcNsdD1/NsdD2-RNAi transformant and mock transformant were grown in PDYA agar medium at 28°C in the dark for 4 days. Mycelia were collected and subsequently frozen in liquid nitrogen. After mRNA purification and library construction, the samples were sequenced by next-generation sequencing (NGS) based on the Illumina sequencing platform. The threshold value of differentially expressed genes was a fold change of >2 and a *P* value of <0.05 (66). RNA isolation, mRNA purification, and cDNA synthesis and sequencing were performed by Oebiotech (Shanghai). All samples were evaluated using three biological replicates.

### Recombinant expression and purification of CcNsdD2-C and preparation of an antibody against CcNsdD2-C.

Recombinant expression and purification of CcNsdD2-C and preparation of an antibody against CcNsdD2-C were performed by the GenScript Company (Nanjing). Briefly, the CcNsdD2-C expression plasmid was constructed by cloning the RT-PCR product encoding the predicted C-terminal DNA-binding domain (residues 480 to 700) of CcNsdD2 (CcNsdD2-C) into pET-30a(+) digested with NdeI and HindIII, creating pET-30a-Cc*nsdD2*-C. pET-30a-Cc*nsdD2*-C was transformed into Escherichia coli BL21(DE3) (TransGen Biotech) to express CcNsdD2-C. The recombinant CcNsdD2-C was purified by Ni-affinity chromatography followed by Superdex 200 molecular size exclusive chromatography to approximately 90% purity. Purified recombinant CcNsdD2-C was injected into New Zealand White rabbits to raise antibodies against CcNsdD2-C (anti-NsdD2-C).

### ChIP-seq and MEME analyses.

Chromatin immunoprecipitation and sequencing (ChIP-seq) were performed by Shanghai Jiayin Biotechnology (Shanghai) as previously described (67, 68). Briefly, the mycelia of the wild-type AmutBmut strain were grown in PDYA agar medium at 28°C in the dark for 4 days. Then, the mycelia were collected, frozen, and ground into fine powders in liquid nitrogen. The sample was cross-linked for 10 min at room temperature with 1% formaldehyde solution, followed by 5 min of quenching with 0.125 M glycine. Then sample was washed twice with cold phosphate-buffered saline (PBS), and the supernatant was aspirated. Isolated nuclei were then sheared in buffer containing 1% SDS, 10 mM EDTA, and 50 mM Tris for 40 cycles (30 s on, 30 s off) using a Diagenode Bioruptor to shear chromatin into 100- to 400-bp fragments. Sonicated lysates were cleared once by centrifugation and incubated overnight at 4°C with magnetic beads bound with anti-NsdD2-C antibody to enrich for DNA fragments bound by CcNsdD2. For the preparation of the magnetic beads bound with anti-NsdD2-C antibody, 70 μl of protein G-Dynabeads (Life Technologies) was blocked with 0.5% (wt/vol) bovine serum albumin in PBS first, and then magnetic beads were bound with 10 μg of anti-NsdD2-C antibody. After an overnight incubation with the cleared sonicated lysates, magnetic beads were washed with radioimmunoprecipitation assay buffer and 1 M NH_4_HCO_3_. DNA was eluted from the magnetic beads with elution buffer. After an overnight incubation for reverse cross-linking, protein in the elution sample was digested using proteinase K, and DNA was purified with a HiPure Gel Pure DNA minikit. Purified ChIP DNA was used to prepare Illumina multiplexed sequencing libraries using an NEB/NEBNext Library Quant kit for Illumina (E7630S). Amplified libraries were size-selected using a 2% gel to capture fragments between 200 and 500 bp. Libraries were quantified by Agilent 2100 and sequenced on the Illumina NovaSeq 6000.

Paired end reads from two independent ChIP-seq experiments were quality checked with FastQC (http://www.bioinformatics.babraham.ac.uk/projects/fastqc/). Before read mapping, clean reads were obtained from the raw reads by removing the adaptor sequences. Paired-end ChIP-seq reads were aligned using BWA-MEM (v.0.7.17) (69) against the *C. cinerea* genome assembly with default settings. PCR duplicates were not present in the data set. Alignments were filtered with SAMtools (v1.3) (69) to exclude reads with a mapping quality of <30. MACS2 callpeak (v2.1.2) (70) was used for peak calling on individual replicates for each ChIP (treatment) and input (control) pair, using a *q* value of <0.05. Peaks were annotated by the function of annotatePeak of ChIPseeker. Reads distributions (from bigwig) across gene are presented as an average plot (average of reads signals across the targeted genes) by the deepTools tool. Results reported are common target sequences from two independent ChIP-seq and the overlapping binding enriching area in the two independent ChIP-seq of the same common target sequence was determined using UpSetR.

To identify conserved CcNsdD2 binding sequences, the output target sequences of CcNsdD2 were analyzed by using the MEME suite (http://meme-suite.org/tools/meme) with the class mode for motif discovery and a site distribution of zero or one occurrence per sequence (“zoops”), and the minimum and maximum widths of the motif were set at 6 and 12, respectively.

### Electrophoretic mobility shift assay.

For preparation of DNA probes, approximately 130 to 140 bp of the target gene promoter sequence containing GATC motifs were first amplified from genomic DNA using the primer pair of EMSAs containing probe primer oligonucleotides (see Table S2). The resulting products were then used as the template to be amplified using a probe primer labeled with Cy5 to generate Cy5-labeled probe DNA (66).

For generation of the mutant probes, the upstream PCR product of 130 to 140 bp of the specific target promoter sequence, which flanks the sequence containing the GATC motif, was amplified from genomic DNA by PCR using a muF1 forward primer containing homologous sequences of the plasmid pCcExp cloning site and a muR1 reverse primer containing the desired mutation sequence ATAA instead of GATC; the downstream PCR product of the specific target promoter sequence was amplified by PCR using a muF2 forward primer containing the desired mutation sequence ATAA and a muR2 reverse primer containing homologous sequences of the pCcExp cloning site. The upstream PCR product and the downstream PCR product of the specific target promoter were directly used for recombination with pCcExp linearized by digestion with NcoI and KpnI at the cloning site to generate specific pCcMutP using a ClonExpress MultiS cloning kit (Vazyme). The mutant sequence was amplified from pCcMutP by PCR and used as a template to generate the Cy5-labeled mutant probe DNA as described above.

For EMSA, 30-μL portions of the reaction mixtures containing 1× EMSA binding buffer, 1.5 μg of salmon sperm DNA, 50 ng of probe DNA or mutant probe DNA, and 4 μg of CcNsdD2-C were incubated at 37°C for 30 min. Then, the sample was separated on a 5% polyacrylamide gel in 1× Tris-borate EDTA buffer (pH 8.3), and the Cy5-labeled probes were detected by an Odyssey machine (LI-COR). Twentyfold unlabeled DNA as a competitive cold probe was added to the reaction mixture when necessary (66).

### Data availability.

The RNA-seq and ChIP-seq data have been deposited in the NCBI Sequence Read Archive under accession numbers PRJNA720013 and PRJNA720896, respectively. Other relevant data supporting the findings of this study are available in this article and its associated supplemental material.
