# Supplementary figures and images for: Molecular Mechanism by Which the GATA Transcription Factor CcNsdD2 Regulates the Developmental Fate of Coprinopsis cinerea under Dark or Light Conditions
Source: mBio. 2022 Feb 1;13(1):e03626-21. doi: 10.1128/mbio.03626-21 (PMC8805025; doi:10.1128/mbio.03626-21)

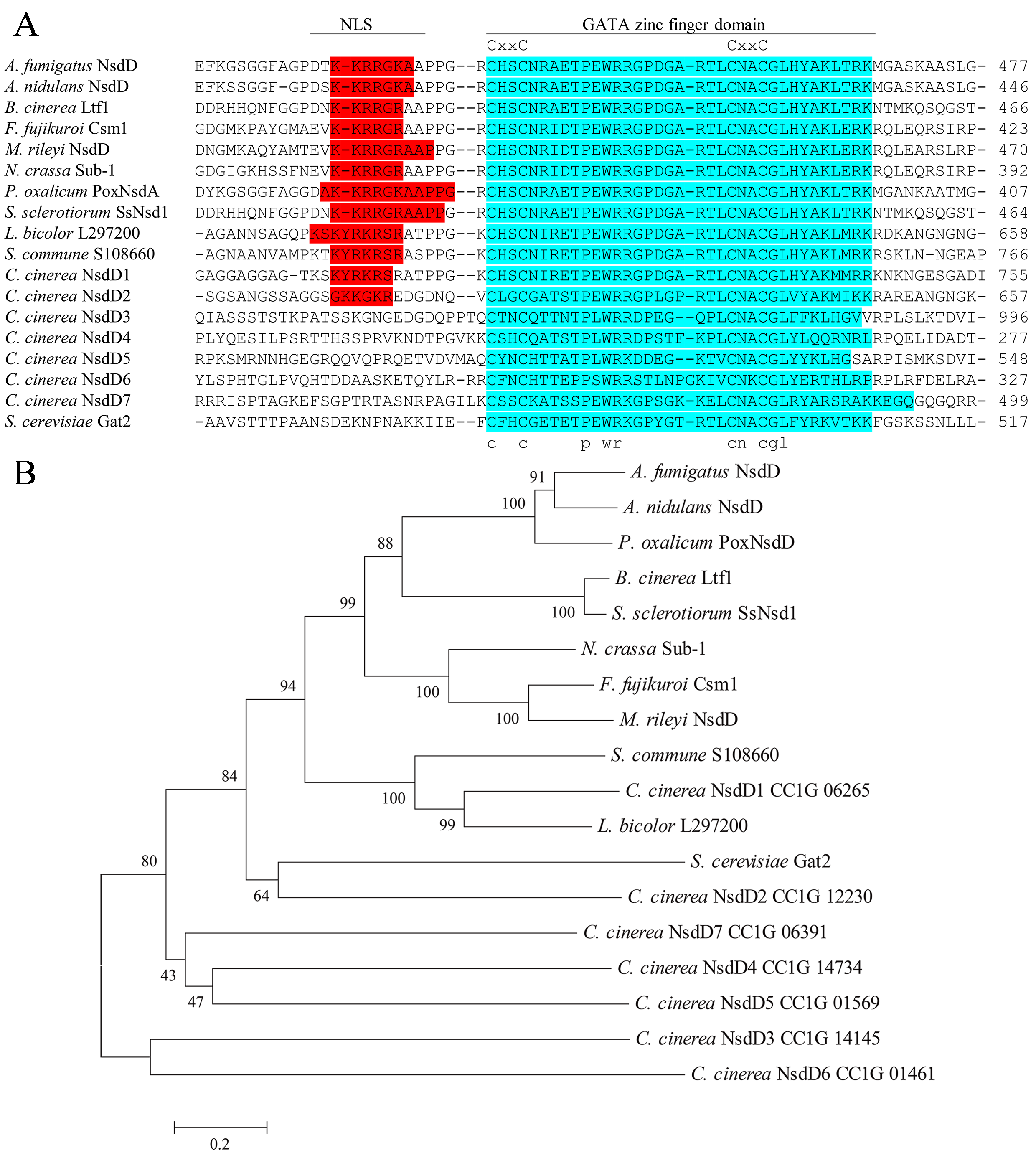

Supplement: FIG S1 [file mbio.03626-21-sf001.tif]

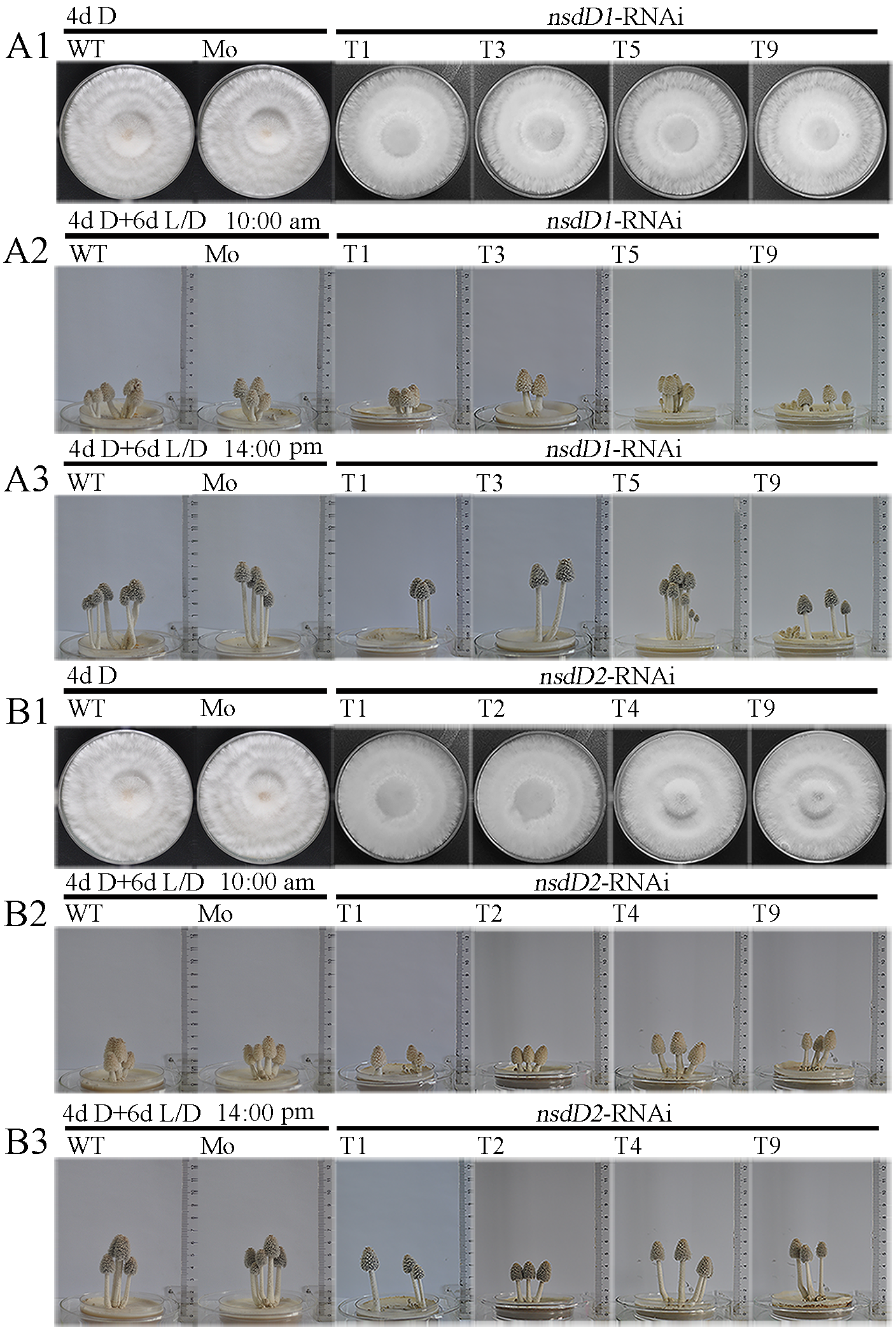

Supplement: FIG S2 [file mbio.03626-21-sf002.tif]

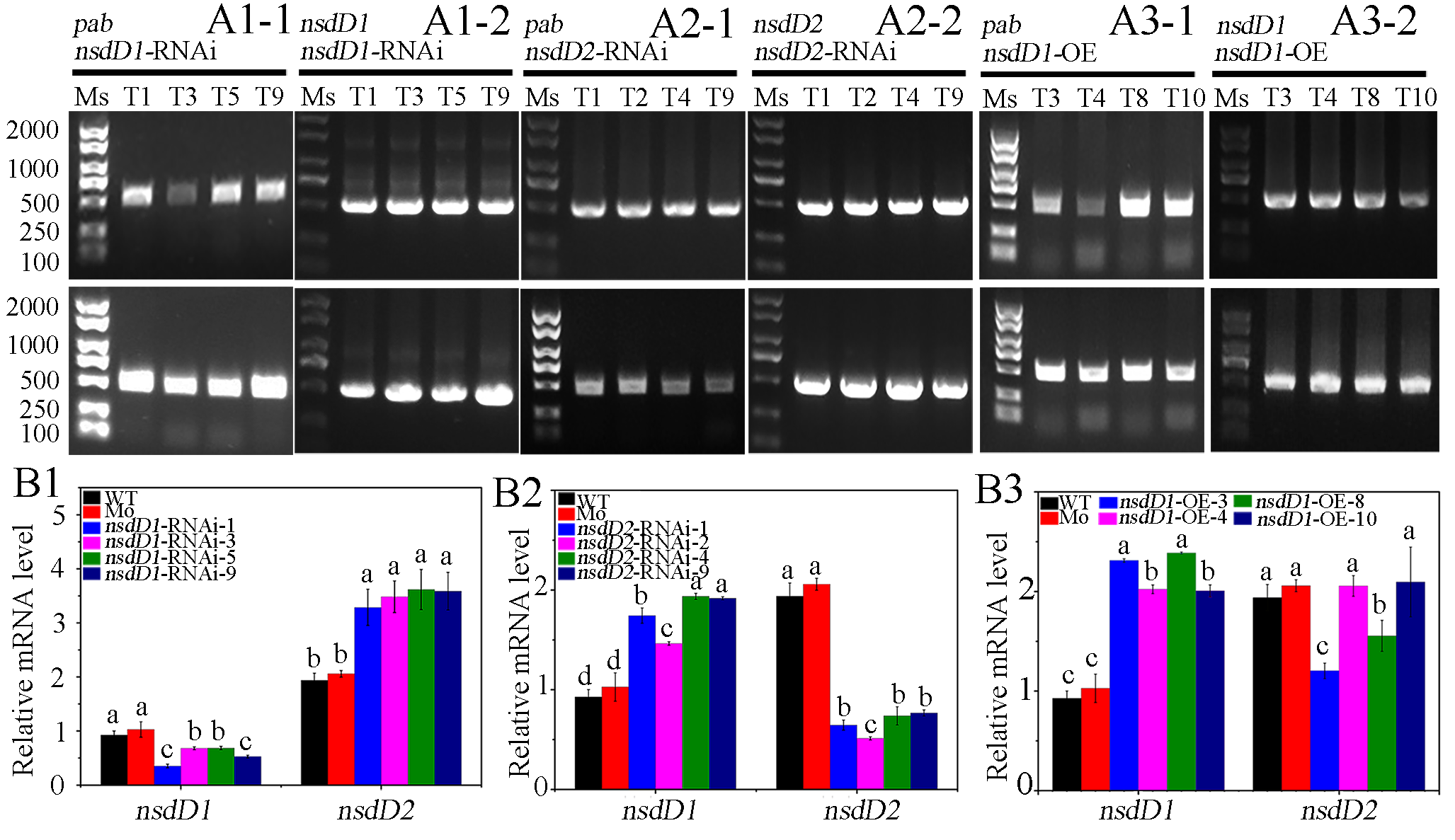

Supplement: FIG S3 [file mbio.03626-21-sf003.tif]

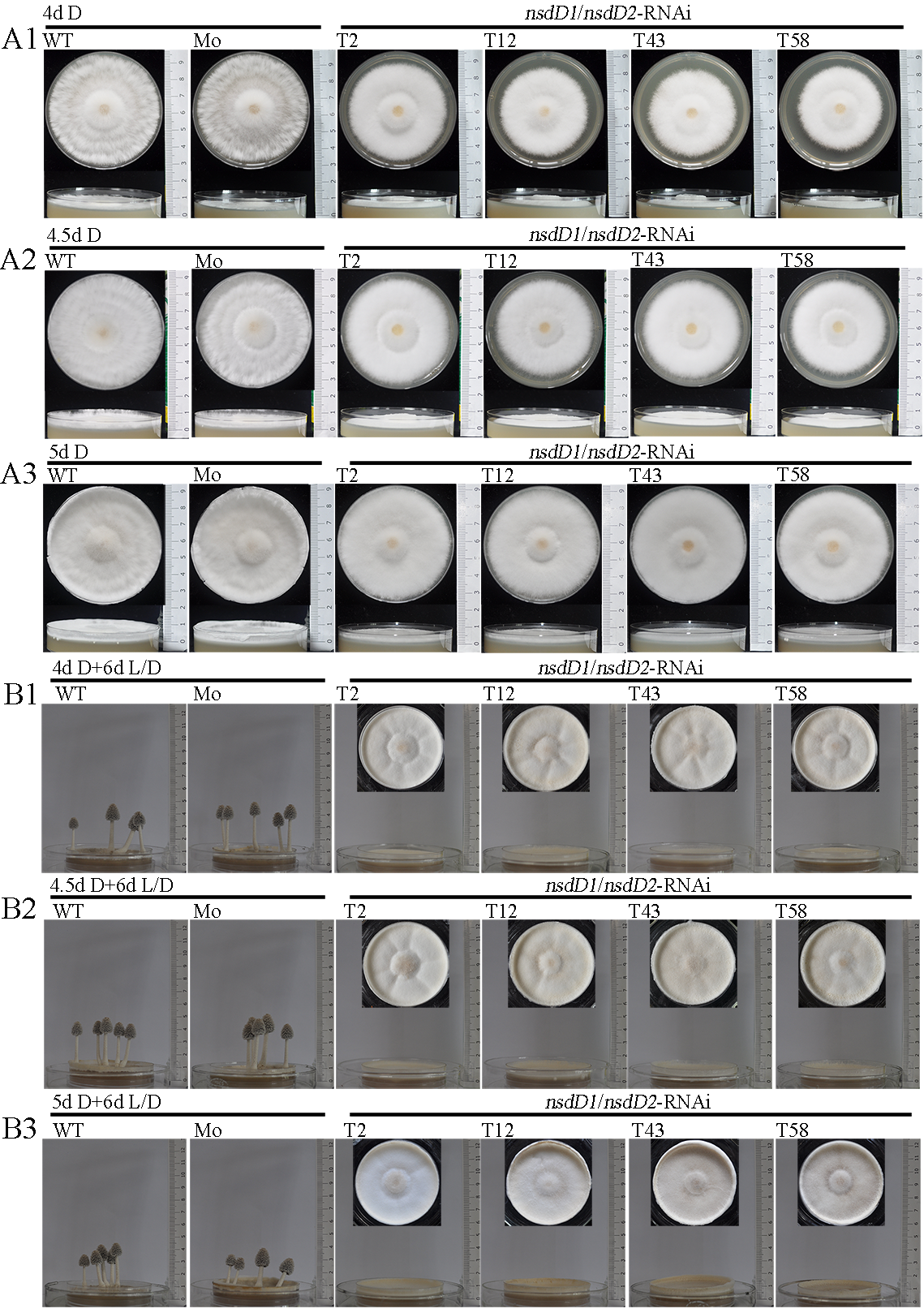

Supplement: FIG S4 [file mbio.03626-21-sf004.tif]

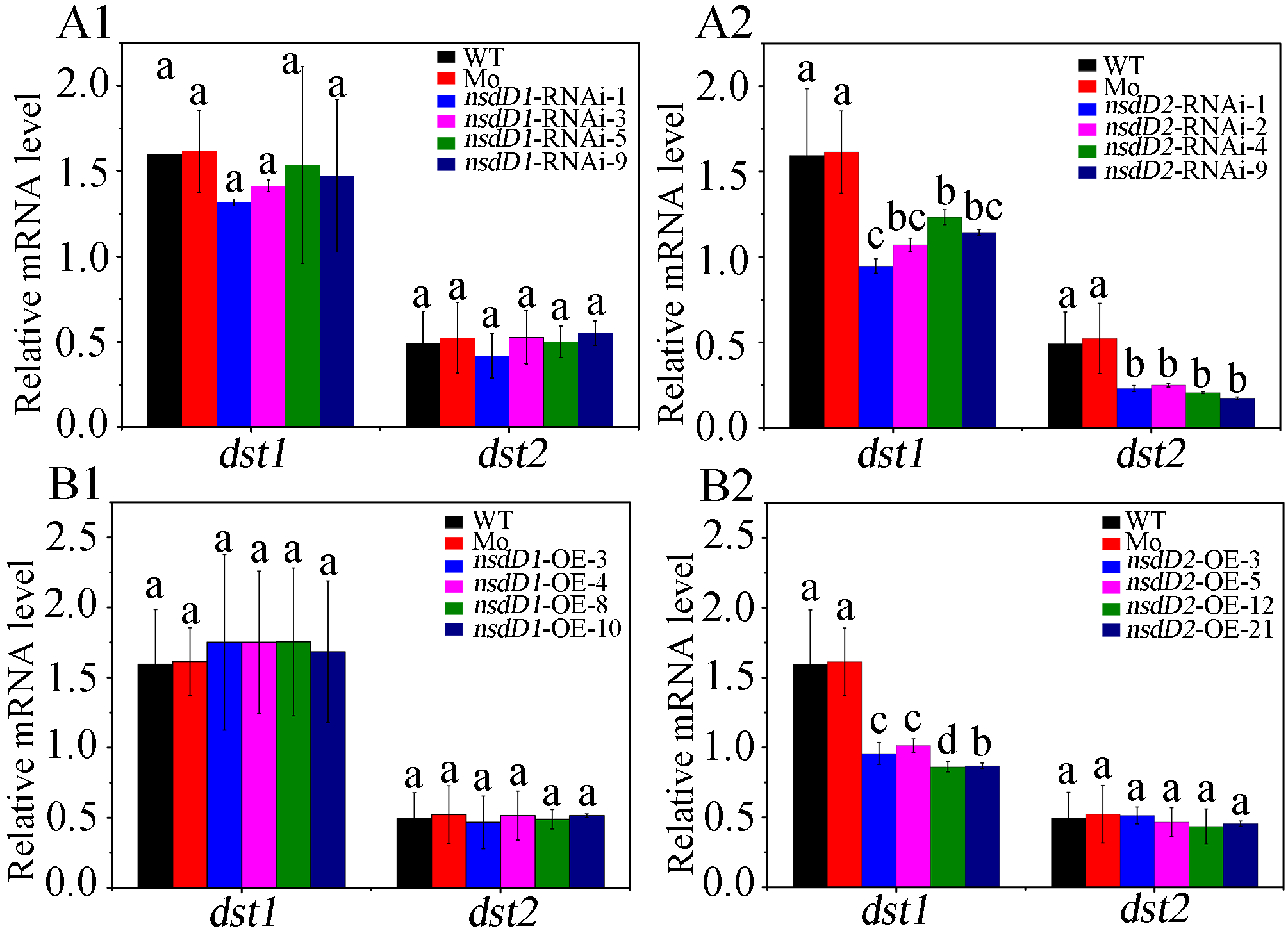

Supplement: FIG S6 [file mbio.03626-21-sf006.tif]

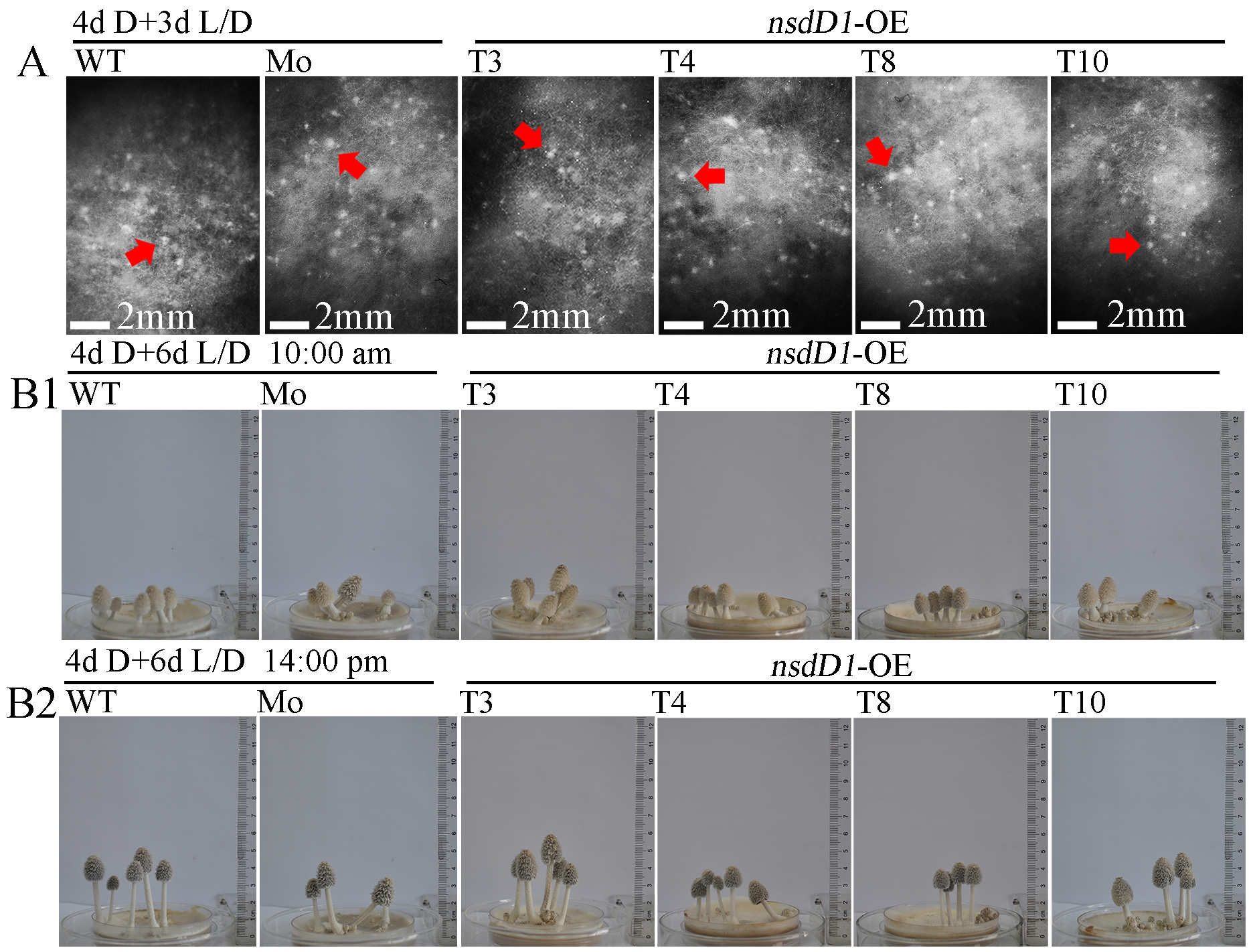

Supplement: FIG S5 [file mbio.03626-21-sf005.tif]
