# Supplementary material for: Molecular Mechanism by Which the GATA Transcription Factor CcNsdD2 Regulates the Developmental Fate of Coprinopsis cinerea under Dark or Light Conditions
Source: mBio. 2022 Feb 1;13(1):e03626-21. doi: 10.1128/mbio.03626-21 (PMC8805025; doi:10.1128/mbio.03626-21)
Supplement: TABLE S1 [file mbio.03626-21-st001.docx]

Table S1. The number of primary hyphal knots, secondary hyphal knots, fruiting bodies and sclerotia produced by wild-type AmutBmut strain, mock transformant and CcNsdD2-OE transformant cultivated under indicated conditions.

| Item | WT | Mo | CcNsdD2-OE (n=3) | | | |
| --- | --- | --- | --- | --- | --- | --- |
|  | (n=12) | (n=12) | T3 | T5 | T12 | T21 |
| Primary hyphal knots^1a^  (No. mm^-2^) | 1.1±0.28^b^ | 1.22±0.26^b^ | 3.09±0.36^a^ | 3.15±0.46^a^ | 3.03±0.57^a^ | 2.67±0.11^a^ |
| Secondary hyphal knots^1b^  (No. mm^-2^) | 0.66±0.05^d^ | 0.67±0.04^d^ | 5.87±0.28^a^ | 5.21±0.21^b^ | 4.73±0.28^c^ | 4.58±0.34^c^ |
| Fruiting bodies^1c^  (No. plate^-1^) | 4.83±1.40^b^ | 4.50±1.62^b^ | 10.67±2.08^a^ | 12.67±1.53^a^ | 15.00±2.00^a^ | 12.33±4.16^a^ |
| Primary hyphal knots^2a^  (No. mm^-2^) | 2.47±0.20^c^ | 2.73±0.22^c^ | 7.82±0.36^b^ | 7.64±0.28^b^ | 8.30±0.28^ab^ | 8.61±0.46^a^ |
| Sclerotia^2b^  (No. mm^-2^) | 12.88±0.64^b^ | 12.90±0.55^b^ | 21.76±0.82^a^ | 21.76±1.41^a^ | 21.39±0.91^a^ | 22.90±0.83^a^ |

1, Cultures were first cultivated in constant darkness for 4 d, and then, were cultivated under a 12 h light/12 h dark rhythm for 1 d (1a), 3 d (1b), or 6 d (1c), as shown in Fig 7A1, A2, and A3.

2, Cultures were first cultivated in constant darkness for 4 d, and then, were cultivated in the darkness for 1 d (2a) or 5 d (2b), as shown in Fig 7B1 and B2.

WT, wild-type parent AmutBmut strain; Mo, mock transformant; T3, T5, T12, and T21, representative CcNsdD2-OE transformants. Values represent the means ± SD (n=3 or 12). The same letters indicate no significant difference (*p* >0.05) and different letters indicate significant differences (*p* <0.001) between the wild-type AmutBmut strain, mock transformant and CcNsdD2-OE transformant by Duncan’s test.
