# Supplementary material for: Molecular Mechanism by Which the GATA Transcription Factor CcNsdD2 Regulates the Developmental Fate of Coprinopsis cinerea under Dark or Light Conditions
Source: mBio. 2022 Feb 1;13(1):e03626-21. doi: 10.1128/mbio.03626-21 (PMC8805025; doi:10.1128/mbio.03626-21)
Supplement: TABLE S2 [file mbio.03626-21-st002.docx]

**Table S2 Primers used in PCR for this sdudy**

| **Name/Purpose** | **Sequence (5’ to 3’)** | | | | |
| --- | --- | --- | --- | --- | --- |
| **Primers used in PCR for construction of gene silencing plasmids** | | | |  | |
| *nsdD1*-AS-F | CACACAACAAGCTCATCGCCCCATGGAGGATTCCAGTGTCCCTGTGGTG | | | | |
| *nsdD1*-AS-R | TCGTTGGCAATACTCCACCCATGGATGGCTTCGACGCATTATCCAAC | | | | |
| *nsdD1*-S-F | TGCACGGGAATATTTCGCGGTACCGCAGCAACGGCAGCTACAGGC | | | | |
| *nsdD1*-S-R | ATCCCGGTCGGCATCTACTGGTACCAGGATTCCAGTGTCCCTGTGGTGG | | | | |
| *nsdD2*-AS-F | CACACAACAAGCTCATCGCCCCATGGAGCAGCCAACGAGCTCGAAGAAC | | | | |
| *nsdD2*-AS-R | TCGTTGGCAATACTCCACCCATGGATGTCATCGGACGGGCGATACA | | | | |
| *nsdD2*-S-F | TGCACGGGAATATTTCGCGGTACCTCCGCAACAAGTTCAAGCCC | | | | |
| *nsdD2*-S-R | ATCCCGGTCGGCATCTACTGGTACCAGCAGCCAACGAGCTCGAAGAAC | | | | |
| **Primers used in genomic PCR for analysis of gene silencing transformants** | | | | | |
| *nsdD1*-up-F | GCGTCATTCTGTGTCAGGCTAGCAG | | | | |
| *nsdD1*-up-R | CAGCAACAACAACATCACCAACAGC | | | | |
| *nsdD1*-down-F | CAACAACAACATCACCAACAGCACC | | | | |
| *nsdD1*-down-R | CGCGTTTTATTCTTGTTGACATGGAG | | | | |
| *nsdD2*-up-F | GCGTCATTCTGTGTCAGGCTAGCAG | | | | |
| *nsdD2*-up-R | CCCGTTGAATCCCACGTACCG | | | | |
| *nsdD2*-down-F | GTTGAATCCCACGTACCGACATACG | | | | |
| *nsdD2*-down-R | CGCGTTTTATTCTTGTTGACATGGAG | | | | |
| *Pab1*-up-F | CAGGAAACAGCTATGACCATGATTACGC | | | | |
| *Pab1*-up-R | GCGTGAATGAGTCGTACGAATCGAC | | | | |
| *Pab1*-down-F | GGTGAGGAAGTTGAGGTCGGTATGG | | | | |
| *Pab1*-down-R | GTAAAACGACGGCCAGTGAATTGTAATAC | | | | |
| pCcExp-up-F | GGGCTGGCTTAACTATGCGGCATC | | | | |
| pCcExp-up-R | AGATGGTGGATGTGACCGGAATTGG | | | | |
| pCcExp-down-F | CGAGACTGAGGAATCCGCTCTTGGC | | | | |
| pCcExp-down-R | TCCGGCTCGTATGTTGTGTGGAATTG | | | | |
| **Primers used in PCR for construction of gene overexpression plasmids** | | | | |  |
| *nsdD1*-OE-F | AACAAGCTCATCGATGGCTTCGACGCATTATCCAAC | | | | |
| *nsdD1*-OE-R | CGGTCGGCATCTACTGGTACCTCACCGATGACGGGCCAAG | | | | |
| *nsdD2*-OE-F | AACAAGCTCATCGATGTCATCGGACGGGCGATAC | | | | |
| *nsdD2*-OE-R | CGGTCGGCATCTACTGGTACCCTATGGCCTAGAGTTGTTGGTGTTGTC | | | | |
| **Primers used in genomic PCR for analysis of overexpression transformants** | | | | | |
| O-*nsdD1*-up-F | GCGTCATTCTGTGTCAGGCTAGCAG | | | | |
| O-*nsdD1*-up-R | TGGTGACTATGCGTCCGTGTCG | | | | |
| O-*nsdD1*-down-F | GGACGAGTTCCAATAACAACAATAATCA | | | | |
| O-*nsdD1*-down-R | CGCGTTTTATTCTTGTTGACATGGAG | | | | |
| O-*nsdD2*-up-F | GCGTCATTCTGTGTCAGGCTAGCAG | | | | |
| O-*nsdD2*-up-R | AGCAGGGGGATACACTGGTTGG | | | | |
| O-*nsdD2*-down-F | GCTTGAGCTTGCTCGTCTGTGC | | | | |
| O-*nsdD2*-down-R | CGCGTTTTATTCTTGTTGACATGGAG | | | | |
| **Primers used in PCR for Southern blot probes** | | |  | | |
| S-gpdII-F | TGCGATGAGGTTGTGTATGTAGCG | | | | |
| S-gpdII-R | GGCGATGAGCTTGTTGTGTGTAGAT | | | | |
| S-*nsdD1*-F | CTATCCACCACAGTACGCTTCCT | | | | |
| S-*nsdD1*-R | AGGATTCCAGTGTCCCTGTGG | | | | |
| S-*nsdD2*-F | TCCGCAACAAGTTCAAGCCC | | | | |
| S-*nsdD2*-R | AGCAGCCAACGAGCTCGAAGA | | | | |
| **Primers used in qRT-PCR** | |  | | | |
| β-tubulin-F | GGAGAGACCTTTTGGGAGATGC | | | | |
| β-tubulin-R | CATGGTCGACTTGGTCGAAATATAC | | | | |
| Q-*nsdD1*-F | CGACAATGCAGGCTATGTGCACC | | | | |
| Q- *nsdD1*-R | GGAAGCGTACTGTGGTGGATAGTTCC | | | | |
| Q- *nsdD2*-F | GCAATCCTCACAATGAAGGCTGGG | | | | |
| Q- *nsdD2*-R | CGAAGAACTGGTCGGAGAAGGGTCT | | | | |
| Q-*cfs1*-F | GCGTATATGATTGGAGATTGCGAGG | | | | |
| Q- *cfs1*-R  Q- *cfs2*-F  Q- *cfs2*-R | GGATGATATCCGTGCAACAGTAGACG  CATGTACGGCGAGGTGGATGTAGAA  GGTGAAGAGGGAGGCGAGGTAGG | | | | |
| Q- *cfs3*-F | GAACGCTATCGCTGAAGTCCCAGAG | | | | |
| Q- *cfs3*-R | CGCCCAGTTGGATATAGACAGCATG | | | | |
| Q-*cgl1*-F | GCGGCTGCGATTGCGTACAG | | | | |
| Q- *cgl1*-R | GTTGACGAAAAGGTGGTAGAGCATCTAAG | | | | |
| Q- *cgl2*-F | CAGATCCGCTTCGACTATGGGACTT | | | | |
| Q- *cgl2*-R  Q- *cgl3*-F  Q- *cgl3*-R | GGAAGTGGGGGGAGCAATCCA  TCGGACAAGCTCGACCTTGAACC  CGTTCTCCCTTCGTCGAATGCC | | | | |
| Q- *ich1*-F | GCTGCTGTTAATCGTATCGTTGGTGC | | | | |
| Q- *ich1*-R  Q-*ich2*-F  Q- *ich2*-R | GCAGCTTCCACCAGGCGCA  CGGGGTTGATGCTAATAAACTCGG  GGGGTTGGTTGAGAGTAACTGCATG | | | | |
| Q- *hyd1*-F | AACAGTGCAACGGTGGCGAGA | | | | |
| Q- *hyd1*-R | ACCCGCAGTGATCTGCTTAACGTC | | | | |
| Q- *hyd2*-F | AGTGCAACGGCGGTGAAGTCC | | | | |
| Q- *hyd2*-R  Q-*hyd3*-F  Q- *hyd3*-R | ACCTGCCCAGTAATCTGCTTGAGATC  GGCCGAAGCTCTAGCCCAAGG  GCGTCCCTATAGAGATTCAGGATGGA | | | | |
| Q-*lcc12*-F | GTCACTCAGTGTCCCATTGTTCCTGG | | | | |
| Q-*lcc12*-R | ACGCAGGCCGTCACAGTATTGG | | | | |
| Q- *lcc16*-F | CGACGCAGCACTGCTATCCATTG | | | | |
| Q- *lcc16*-R  Q-*lcc7*-F  Q-*lcc7*-R  Q-*dst1*-F  Q-*dst1*-R  Q-*dst2*-F  Q-*dst2*-R | GGACTGTGTATTCAGCCGTTGAGTTGA  TCTTCAGCTTGCCATCGTGCTCTAC  CCTGGGTGGACGCCATTGACTAC  CTGGCGCATTGATGCAAACTCAG  CCATCTCCCATTTGGCTCCCTTC  CGACCTCAGCAAGATCGTGGAGG  GCGACGCTTACCGCTGCTGAT | | | | |
| **Primers used in PCR for DNA probes in EMSA** | | | | | |
| Cy5-labelled | Cy5-AGCACGTGGTCGAAAG | | | | |
| E-*cfs1*-F | AGCACGTGGTCGAAAGTAACCAAAGCGCGCGATGC | | | | |
| E- *cfs1*-R | AGCACGTGGTCGAAAGCCGTGGTTGGGCAGATGTGG | | | | |
| E- *cfs2*-F | AGCACGTGGTCGAAAGGAAGTTGCTGTATACTCGATGACA | | | | |
| E- *cfs2*-R | AGCACGTGGTCGAAAGATCTCCATCTTATCTACTCGTTGTT | | | | |
| E-*cgl1*-F | AGCACGTGGTCGAAAGGATTCGGCTCTTCCGACTTCATCTC | | | | |
| E- *cgl1*-R | AGCACGTGGTCGAAAGTTCGCAATTTCATTGCATCCCTG | | | | |
| E- *cgl3*-F | AGCACGTGGTCGAAAGAACGAGCTCCCATGCGAGAAAG | | | | |
| E- *cgl3*-R | AGCACGTGGTCGAAAGGCGAGACGGTGCAGAGAGTGTG | | | | |
| E-*hyd1*-F | AGCACGTGGTCGAAAGCTCCTGAGCTGTGACTGTATTCCTT | | | | |
| E- *hyd1*-R  E-*nsdD1*-F  E- *nsdD1*-R  E-*dst1*-F  E-*dst1*-R | AGCACGTGGTCGAAAGCGTACTGTGGAGCAGGGAGAAC  AGCACGTGGTCGAAAGTCAGACGAAATCGCCGATTC  AGCACGTGGTCGAAAGGAGCCGTTGGATAATGCGTC  AGCACGTGGTCGAAAGCCGCACTACACTCTCCGAGGC AGCACGTGGTCGAAAGTGGTTACTTTAACTAAATTCAGGGCAC | | | | |
| Mu-*cfs1*-F1 | CACACAACAAGCTCATCGCCTTGGGCAATATGGATGGGAA | | | | |
| Mu-*cfs1*-R1 | GGGGGCAAATATTATGCGTAGGAGCAAAAGGCGT | | | | |
| Mu-*cfs1*-F2 | CGCATAATATTTGCCCCCTTCGCGAATCCGTGA | | | | |
| Mu-*cfs1*-R2 | ATCCCGGTCGGCATCTACTTTGATGATCTCCTTCTGAATCGGAA | | | | |
| Mu-*cfs2*-F1 | CACACAACAAGCTCATCGCCGTCTTCACTTGAGGTGCAGCTTCCCT | | | | |
| Mu-*cfs2*-R1 | CGCTTTTTTATTCCGCTTGAAACTCTCACGGGC | | | | |
| Mu-*cfs2*-F2 | AAGCGGAATAAAAAAGCGATACAGTGATTAGATTAAAACA | | | | |
| Mu-*cfs2*-R2 | ATCCCGGTCGGCATCTACTTGATCTCCTTCTGAATCGGAAATG | | | | |
| Mu-*cgl1*-F1 | CACACAACAAGCTCATCGCCTTTGCTTACTGTTACTGGATGCGAA | | | | |
| Mu-*cgl1*-R1 | TGATATTATGGCATCCCCAATGACGGAGATGAA | | | | |
| Mu-*cgl1*-F2 | GGGGATGCCATAATATCAAGCTAACTCTGGTCATATCTGG | | | | |
| Mu-*cgl1*-R2 | ATCCCGGTCGGCATCTACTCATACGGGACACGTTCCTCG | | | | |
| Mu-*cgl3*-F1 | CACACAACAAGCTCATCGCCCGCTCTGCACCGAAGCACT | | | | |
| Mu-*cgl3*-R1 | CGAGAATGAAATTATTACATGTGAAATCCACAGACCTCAG | | | | |
| Mu-*cgl3*-F2 | GTAATAATTTCATTCTCGAAGCTCTCGTATGCA | | | | |
| Mu-*cgl3*-R2 | ATCCCGGTCGGCATCTACTCCCTGAGCTGCCTCCTATACGC | | | | |
| Mu-*hyd1*-F1 | CACACAACAAGCTCATCGCCAGAAATAAAGCCCTGGCCCAC | | | | |
| Mu-*hyd1*-R1 | TCAAGCATTATGGTGTACAGATATAGAATGTTTAGCTTGTT | | | | |
| Mu-*hyd1*-F2 | GTACACCATAATGCTTGATTTGGTCTAGCCGAT | | | | |
| Mu-*hyd1*-R2  Mu-*nsdd1*-F1  Mu-*nsdd1*-R1  Mu-*nsdd1*-F2  Mu-*nsdd1*-R2  Mu-*dst1*-F1  Mu-*dst1*-R1  Mu-*dst1*-F2  Mu-*dst1*-R2 | ATCCCGGTCGGCATCTACTCTTAACGTCAATGTTCAACAGGCC  CACACAACAAGCTCATCGCCAGTGGTGGGATGGGTGCGAGA  GATTATTATCGGTGAGCGACCCACCGAGATCGG  CGCTCACCGATAATAATCAAAACGATTTCGTTTCTGTGA  ATCCCGGTCGGCATCTACTGGAAGCGTACTGTGGTGGATAGTTCTG  CACACAACAAGCTCATCGCCTAGGGATGGTGCCTACGTGCAA  CGATCTCGTTTATATCACACAAAATTCTGACGTCATTAA  GTGATATAAACGAGATCGACACAAGGCTTGGGT  ATCCCGGTCGGCATCTACTCCAACGTACGGTTCGTCTTGATATAC | | | | |
